# A single-arm, open-label, phase 2 clinical trial evaluating disease response following treatment with BI-505, a human anti-intercellular adhesion molecule-1 monoclonal antibody, in patients with smoldering multiple myeloma

**DOI:** 10.1371/journal.pone.0171205

**Published:** 2017-02-03

**Authors:** Stina Wichert, Gunnar Juliusson, Åsa Johansson, Elisabeth Sonesson, Ingrid Teige, Anna Teige Wickenberg, Björn Frendeus, Magnus Korsgren, Markus Hansson

**Affiliations:** 1 Department of Hematology, Skåne University Hospital and Lund University, Lund, Sweden; 2 Clinical Immunology and Transfusion Medicine, University and Regional Laboratories Region Skåne, Lund, Sweden; 3 Clinical Development, BioInvent International AB, Lund, Sweden; 4 Research, BioInvent International AB, Lund, Sweden; Cardiff University, UNITED KINGDOM

## Abstract

**Background:**

Smoldering multiple myeloma (SMM) is an indolent disease stage, considered to represent the transition phase from the premalignant MGUS (Monoclonal Gammopathy of Undetermined Significance) state towards symptomatic multiple myeloma (MM). Even though this diagnosis provides an opportunity for early intervention, few treatment studies have been done and the current standard of care is observation until progression. BI-505, a monoclonal antibody directed against intercellular adhesion molecule 1 (ICAM-1) with promising anti-myeloma activity in preclinical trials, is a possible treatment approach for this patient category with potential to eliminate tumor cells with minimal long-term side effects. BI-505 was well tolerated in an earlier phase 1 trial.

**Methods and findings:**

In this phase 2 trial the effects of BI-505 in patients with SMM were studied. Four patients were enrolled and three of them completed the first cycle of treatment defined as 5 doses of BI-505, a total of 43 mg/kg BW, over a 7-week period. In the three evaluable patients, BI-505 showed a benign safety profile. None of the patients achieved a response as defined per protocol. EudraCT number: 2012-004884-29.

**Conclusions:**

The study was conducted to assess the efficacy, safety and pharmacodynamics of BI-505 in patients with SMM. BI-505 showed no clinically relevant efficacy on disease activity in these patients with SMM, even if well tolerated.

**Trial registration:**

ClinicalTrials.gov Identifier: NCT01838369.

## Introduction

Smoldering multiple myeloma (SMM), first described as a clinical entity by Kyle and Greipp [1] in 1980, accounts for 10 to 15% of all myeloma diagnoses [2]. It is an asymptomatic plasma cell dyscrasia, with the risk of progression to symptomatic multiple myeloma or systemic amyloidosis of 10% per year during the first 5 years, after that, the risk of progression decrease to 3% per year for the next 5 years and to 1% per year beyond 10 years of follow-up [3]. The International Myeloma Working Group (IMWG) has defined smoldering multiple myeloma as a disorder where the patient has a serum monoclonal (M) protein (IgG or IgA) ≥3 g/100 ml and/or clonal bone marrow plasma cells (BMPCs) of ≥10%, but no CRAB symptoms (increased **c**alcium level, **r**enal failure, **a**nemia or destructive **b**one lesions) [4]. The current standard of care is observation without treatment until progression to symptomatic multiple myeloma [5]. Only one clinical trial so far has shown a benefit in overall survival for high-risk SMM patients on treatment with lenalidomide/dexamethasone compared to observation [6]. The IMWG criteria has recently been updated [7], allowing, in addition to the classical CRAB symptoms, presence of one of the following three “myeloma defining events (MDEs)”: BMPCs ≥ 60%, serum involved/uninvolved free light chain ratio ≥ 100 or > 1 focal lesions on MRI, to be sufficient for a diagnosis of symptomatic multiple myeloma, implying the possibility of early treatment, before irreversible end-organ damage has occurred.

The cell-surface receptor, intercellular adhesion molecule 1 (ICAM-1), is a transmembrane glycoprotein, which is constitutively expressed at low levels on many cell types including different leukocytes subsets and endothelial cells [8]. Up-regulation of ICAM-1 on endothelial cells at sites of inflammation cause increased leukocyte adhesion with subsequent extravasation and tissue infiltration [9]. In multiple myeloma, the bone marrow microenvironment and adhesion of multiple myeloma cells to bone marrow stroma cells (BMSCs) is essential for tumor cells growth and survival [10]. Adhesion molecules like ICAM-1 on multiple myeloma cells increase the binding capacity to BMSCs and overexpression of ICAM-1 have been associated with more advanced disease and drug resistance [11–13]. Since several independent observations indicate that ICAM-1 is highly expressed and involved in the pathogenesis of myeloma, it constitutes an attractive novel target for immunotherapy of multiple myeloma [14–16].

BI-505 is a fully human, high-affinity IgG1 monoclonal antibody directed against ICAM-1. The BI-505 epitope was strongly expressed on myeloma cells from both newly diagnosed and relapsed patients [16] and the anti-myeloma activity of BI-505 has been evaluated in animal models representing early as well as late-stage disease [16]. In vitro and in vivo mode-of-action studies provide strong evidence for Fc:FcgR-dependent antitumor mechanisms, e.g., macrophage-mediated antibody-dependent cellular phagocytosis and FcγR cross-linking-induced antibody tumor programed cell death, underlying BI-505’s therapeutic activity [16].

A phase 1, multicenter, open-label, nonrandomized, repeat-dose, dose-escalation study of BI-505 in patients with relapsed/refractory multiple myeloma has been conducted [17]. The study was a first-in-human trial to evaluate the tolerability, safety, pharmacodynamics (PD) and pharmacokinetics (PK) of BI-505 following intravenous administration at doses of 0.0004–20.0 mg/kg once every second week. Thirty-four patients were treated with BI-505. The drug was generally well tolerated. Only 3 patients withdrew because of treatment-emergent adverse events (AEs).

In this phase 2 trial the effects of BI-505 in patients with SMM were studied. The primary purpose of this pilot study was to evaluate anti-tumor effect before promoting a large randomized trial. This patient category, untreated and at an early stage of the disease, may be particularly suitable for treatment with monoclonal antibodies, since the therapeutic effects are dependent on a functional immune system, including competent effector cells, such as natural killer cells and macrophages. More advanced disease is characterized by profound immune dysfunction and effective therapies at these stages is likely to require a potential to restore the immune response against myeloma [18, 19]. Moreover, monoclonal antibodies, such as BI-505, offers the possibility of targeted therapy to selectively eliminate tumor cells with minimal side effects, which is preferable for patients with SMM, in whom severe side effects are not acceptable from a risk-benefit perspective.

## Methods

### Study design

This was a single-arm, open-label, phase 2 study designed to assess the disease response following treatment with the monoclonal human antibody BI-505, when administered to patients with SMM. The sample size, 4 to 10 patients, was estimated to minimize the number of patients exposed to BI-505 while obtaining sufficient information to assess effects on disease activity, measured as M protein levels in plasma and urine, and change in percentage of BMPCs.

Following a screening period of up to 14 days, eligible patients were to receive 5 intravenous infusions of BI-505 over a 7-week period: 3 mg/kg body weight (BW) BI-505 on treatment day 1, 10 mg/kg BW BI-505 on treatment day 8 and then 3 infusions of 10 mg/kg BW BI-505 every second week. This was considered 1 dosing cycle of 50 days (Cycle 1).

Patients were evaluated for disease activity based on data collected on treatment day 50. Patients with at least a minimal response (MR) based on M protein levels were allowed to continue to cycle 2, which was to comprise 3 bi-weekly intravenous doses of BI-505 (10 mg/kg BW). Patients who completed Cycle 2 and had at least a partial response (PR) on treatment day 92 were allowed to continue to cycle 3, which was to comprise 3 additional bi-weekly intravenous doses of BI-505 (10 mg/kg BW).

Disease activity, measured as M protein levels in serum/urine, was to be determined throughout the study and was evaluated according to the EBMT criteria [20]. Minor response (MR) was defined as a 25 to 49% reduction in serum M protein level.

The dosing regimen selected for this study was supported by safety, PK and receptor saturation data from the phase 1 study in patients with relapsed/refractory multiple myeloma [17]. In order to reduce the risk of infusion-related reactions observed in the phase 1 trial, the initial dose (3 mg/kg) was lower than subsequent doses (10 mg/kg). Furthermore, the first dose and second doses were administered over a longer time period compared to subsequent doses (4 hours vs. 2 hours). All patients remained at the clinical unit for 10 hours after the start of the first and second infusions and were closely monitored for any untoward reactions. For subsequent infusions, patients were observed for at least 3 hours after the start of the infusion. All patients received paracetamol and an antihistamine (cetirizine) before each infusion.

Based on data from the phase 1 study, where the full PK profile of BI-505 was assessed, dosing with 3 mg/kg BW BI-505 would lead to complete saturation of all ICAM-1 epitopes on the patient’s bone marrow myeloma cells during the first dosing interval (1 week) [17]. Similarly, dosing with 10 mg/kg BW BI-505 would lead to complete saturation during the subsequent 2-week dosing intervals, with trough plasma concentrations above 1 μg/ml, which is needed for full receptor saturation in the bone marrow.

The study protocol, patient information and consent form were reviewed and approved by an Independent Ethics Committee, The Regional Ethical Review Board in Lund, Sweden (www.epn.se), prior to inclusion of patients. The study was conducted in compliance with the protocol, regulatory requirements, good clinical practice (GCP) and the ethical principles of the latest revision of the Declaration of Helsinki as adopted by the World Medical Association. Written informed consent was obtained from all patients before enrollment. EudraCT number: 2012-004884-29. ClinicalTrials.gov Identifier: NCT01838369.

### Patient population

The study included patients ≥ 18 years old with diagnosis of SMM based on the 2009 IMWG criteria [4], i.e.; serum M protein level ≥3 g/100 ml and/or BMPCs of ≥10% and absence of end-organ damage such as lytic bone lesions, anemia, hypercalcemia and renal failure that could be attributed to a plasma cell disorder. All patients had measurable disease, defined by a serum M protein ≥1 g/100 ml, Eastern Cooperative Oncology Group (ECOG) performance status 0–1, adequate renal and hepatic function. Systemic corticosteroid use was not allowed within 4 weeks prior to screening. Patients were excluded if they had clinical suspicion of progression to symptomatic multiple myeloma, if they had any prior or current treatment with proven or potential impact on plasma cell proliferation and survival, or had used any investigational agent within the last 3 months. They were also excluded if clinical findings indicating cardiac or renal amyloid light-chain (AL) amyloidosis (Additional details are provided in S1 Appendix).

### Safety and efficacy assessments

Adverse events (AEs) were assessed according to the National Cancer Institute Common Terminology Criteria for Adverse Events version 4.0. Other safety evaluations were physical examinations including Eastern Cooperative Oncology Group (ECOG) performance status, vital signs, electrocardiograms and laboratory tests (including complete blood count, clinical chemistry, coagulation, and urinalysis).

The primary objective was to assess the tumor response rate, defined according to the EBMT response criteria [20], which included measurements of serum and urine M-protein, serum free light chains, and plasma cell levels in bone marrow. The secondary objectives were to further assess the clinical safety of BI-505 and the pharmacodynamics (PD).

### Pharmacodynamic assessment in blood

The proportion of monocytes, NK and NKT cells in blood samples, collected immediately before and after each BI-505 infusion, was analysed using flow cytometry with monoclonal fluorescent-labelled antibodies with panels including CD14, CD3 and CD56.

### Statistical analysis

There was no statistical analysis. All patients and results are listed.

## Results

### Patients’ characteristics

**Fig 1:**
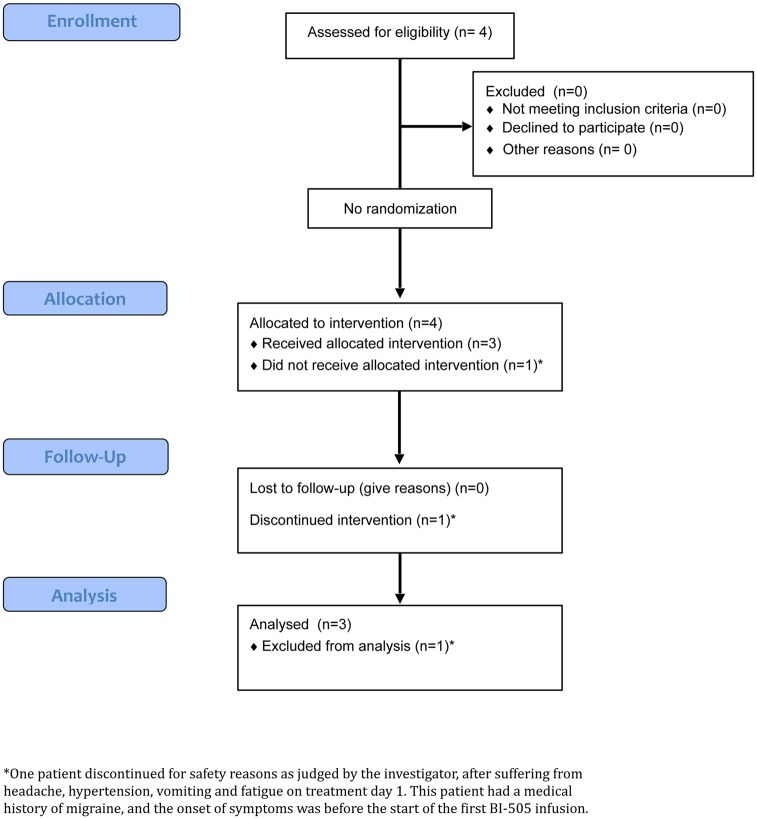
CONSORT flowchart for A single-arm, open-label, phase 2 clinical trial with BI-505, a human anti-intercellular adhesion molecule-1 monoclonal antibody, in patients with smoldering multiple myeloma.

The study was conducted between April 2013 and December 2014 at the Department of Hematology, Skåne University Hospital in Lund, Sweden. Four patients were screened. Bone marrow aspirate smear from the first patient showed at screening 8% BMPCs. The actual sample was assessed to be somewhat diluted by peripheral blood. However, a previous investigation at diagnosis had shown 20% BMPCs and the SMM diagnosis considered confirmed. All four patients enrolled in the study fulfilled the inclusion and exclusion criteria. All patients had an ECOG 0. The ages of the patients were 50, 64, 66 and 67 years (Table 1). Three were male and one was female. In three patients the immunoglobulin was IgG; in one patient it was IgA. The light chain was kappa in three patients and lambda in one patient. A CONSORT flowchart is provided in (Fig 1).

**Table 1:** Patients’ characteristics and efficacy essessments.

Pat No	Sex	Age	M- Comp Class	Diagnosis SMM yr. before inclusion	Serum M protein at diagnosis g/100 ml	Serum M protein Day 1 g/100 ml	Serum M protein Day 50 g/100 ml	Urinary M protein at screening mg/24 h	Urinary M protein Day 50 mg/24 h	BMPCs (%) at screening	BMPCs (%) Day 50 resp. Day 69
**1**	**M**	**66**	**A kappa**	**2.8**	**1.9**	**1.8**	**1.9**	**78**	**62**	**8**	**12**
**2**	**M**	**64**	**G kappa**	**2.3**	**0.7**	**0.9**	**0.9**	**497**	**664**	**17**	**9**
**3**	**M**	**67**	**G lambda**	**2.5**	**2.8**	**3.0**	**3.6**	**18**	**0**	**11**	
**4**	**F**	**50**	**G kappa**	**8.4**	**2.0**	**2.2**				**11**	

The three patients who completed the first dosing cycle received a dose of 2.25 to 2.98 mg/kg BW on treatment day 1 and a dose of 10.00 mg/kg BW on treatment day 8, 22, 36 and 50.

One patient discontinued for safety reasons as judged by the investigator, after suffering from headache, hypertension, vomiting and fatigue on treatment day 1. This was a patient with a medical history of migraine, and onset of symptoms before start of the first BI-505 infusion. These 4 AEs were all judged to be unrelated to the study drug. This patient received a dose of 0.27 mg/kg BW on treatment day 1 and no further doses. The infusion was stopped after 1 hour because of hypertension. The headache and vomiting were resolved on the same day they developed; the fatigue was resolved the day after it developed; and the hypertension was resolved after 136 days.

The Sponsor terminated the trial due to re-assessment of the developmental path for BI-505, focusing on maintenance after high dose melphalan and autologous stem cell transplantation.

### Efficacy assessments

Three patients were assessed for tumor response based on change in serum M protein level on treatment day 50 (Table 1). None of these patients achieved MR, a requirement for continuation to Cycle 2. Between treatment day 1 and 50 (after 4 doses of BI-505), there were no clinically significant changes in serum M protein level; 1.8 to 1.9 g/100 ml, 3.0 to 3.6 g/100 ml and 0.9 to 0.9g/100 ml.

There were no clinically significant changes in twenty-four hour urinary M protein production between screening and treatment day 50 (Table 1); 78 mg to 62 mg, 497 mg to 664 mg and 18 mg to 0 mg.

Change in percentage of BMPCs was assessed in two patients (Table 1). The percentage of BMPCs was 8% at screening and 12% on treatment day 50 for the first patient and 17% at screening and 9% at the End of Study Visit on day 69 for the second patient. For the other two patients, the percentage of BMPCs was only assessed at screening.

### Bone marrow concentrations of BI-505

BI-505 concentration in bone marrow was assessed as previously described [17]. For the three patients assessed, BI-505 concentrations of 12.9 μg/ml (day 50), 21.6 μg/ml (day 69) and 66.1 μg/ml (day 50) were detected in bone marrow aspirates. This indicated complete saturation of ICAM-1, as saturation occurs at a BI-505 concentration of 1 to 3 μg/ml [17].

### Adverse events

A total of 22 AEs were reported in the four patients and 10 of them were judged to have a “Probable/Certain” relationship to the study drug (Table 2); 9 infusion-related reactions with pyrexia, chills and body ache and one case of C-reactive protein increase. One other AE, prolonged symptoms of bronchitis after a common cold, was judged to have a “Possible” relationship to the study drug. Most of the reported AEs were mild. One patient was discontinued after developing AEs (judged to be unrelated to the study drug) on treatment day 1 as described above.

**Table 2:** Adverse events.

Adverse Events	n	Severity	Relationship to Study Treatment
**Overall, treatment-related AEs**	**11**	**Grade 1: (9) Grade 2: (2)**	**Probable/Certain (10) Possible (1)**
**Infusion-related reactions; pyrexia, chills, body ache**	**9**	**Grade 1: (7) Grade 2: (2)**	**Probable/Certain**
**C-reactive protein increase**	**1**	**Grade 1: Mild**	**Probable/Certain**
**Bronchitis**	**1**	**Grade 1: Mild**	**Possible**
**Nasopharyngitis/viral infection**	**1**	**Grade 2**	**Unlikely**
**Headache**	**2**	**Grade 1–2**	**Unrelated**
**Hypertension**	**2**	**Grade 1–2**	**Unrelated**
**Fatigue**	**1**	**Grade 1: Mild**	**Unrelated**
**Vomiting**	**1**	**Grade 1: Mild**	**Unrelated**
**Insomnia**	**1**	**Grade 1: Mild**	**Unrelated**
**Nicotine dependence**	**1**	**Grade 2: Moderate**	**Unrelated**
**Blood cholesterol increase**	**1**	**Grade 1: Mild**	**Unrelated**
**Acute coronary syndrome**	**1**	**Grade 3: Severe**	**Unrelated**

One AE, acute coronary syndrome that occurred one month after the last dose of BI-505, was judged to be serious. This serious adverse event (SAE, Grade 3) was treated by percutaneous transluminal coronary angioplasty. The relationship to the study drug was judged as “Unlikely.”

### Pharmacodynamic assessment in blood

**Fig 2:**
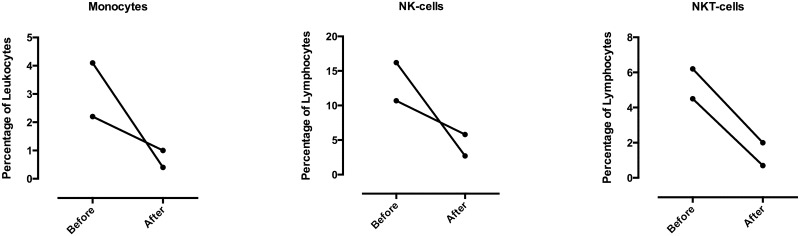
Reduced proportions of monocytes, NK and NKT cells after the first dose of BI-505. Percentage of monocytes, NK and NKT cells in blood samples collected before and immediately after completion of the first BI-505 infusion in patients No 2 and No 3, as analyzed by flow cytometry.

For two patients, blood samples for FACS-analysis were obtained immediately before and after each infusion for exploratory correlative analysis. The proportions of monocytes, NK and NKT cells in peripheral blood after completion of the first infusion (Fig 2), were shown to be reduced. This could be an indirect sign of activated immune response against target cells for BI-505. However, the levels recovered before the second infusion and further decrease was not seen in subsequent infusions (data not shown).

## Discussion

Few treatment studies, aiming to prevent or delay the development of manifest disease in SMM, have been conducted [6, 21–31]. Up to the present, standard of care is close observation without treatment until progression [5]. Theoretically, targeted therapy with monoclonal antibodies, without expected long-term side effects, would be an attractive treatment strategy for patients with SMM. BI-505 is a human monoclonal IgG1 antibody with high specificity for ICAM-1, selected by functional screening for its ability to induce dose-dependent programmed cell death in B lymphoma cell lines [32] and with promising anti-myeloma activity in several murine myeloma models including hu-SCID mice with human fetal bone chip and human primary myeloma cells [15, 16].

Previous clinical trials, with a murine IgG2a anti-ICAM-1 antibody, targeting another ICAM-1 epitope than BI-505, enlimomab, have been performed, with the aim of reducing detrimental inflammatory activity in ischemic stroke, rheumatoid arthritis and burn injuries [33–36]. Altogether, more than 400 patients have received treatment with the murine anti-ICAM-1 antibody in these trials. The randomized stroke trial did not show any benefit for treatment with enlimomab and treated patients experienced more adverse events, mainly fever and pneumonia. The results of the other trials were cautiously positive and the treatment well tolerated. A follow-up study with a second treatment course of enlimomab in patients with rheumatoid arthritis was associated with cases of serum sickness-like reactions [37], probably due to complement-activating properties of murine monoclonal antibodies of IgG2a isotype and the murine nature of the antibody [38]. In contrary, BI-505 is a fully human antibody of IgG1 isotype with less complement activating properties. In pre-clinical studies, BI-505 did not show any induction of apoptosis in resting or activated normal ICAM-1 expressing peripheral blood B cells or human microvascular endothelial cells [16]. Moreover, BI-505 added in solution did not induce cytokine release from peripheral blood mononuclear cells or T cell proliferation, which otherwise might contribute to adverse events to antibody therapy [16].

A first-in-human, phase 1, study of BI-505 in patients with advanced relapsed/refractory multiple myeloma has shown good tolerability, with doses up to 20 mg/kg, the maximum tolerated dose was not reached [17]. An optimal dose, used in the current study, was determined to be 10 mg/kg every two weeks, which resulted in complete saturation of ICAM-1 epitopes on BMPCs during the entire dosing interval [17]. In the heavily pretreated patient cohort in the phase 1 study, no objective responses were observed, but 24% (7/29) achieved stable disease for more than 8 weeks [17].

This study was conducted to assess the efficacy, safety and pharmacodynamics of BI-505 in patients with SMM. The aim was to enroll four to ten patients. Four patients were enrolled and three of them completed the first cycle of treatment, defined as 5 doses of BI-505 (a total of 43 mg/kg BW) over a 7-week period. All three patients who received treatment doses of BI-505 experienced, despite pre-medication, infusion-related adverse events with fever, chills and body pain during the 1^st^, the 1^st^ and 2^nd^, respectively the 1^st^ to 4^th^ infusion, causing transient discomfort, although classified no worse than mild to moderate. One of the patients had after the 2^nd^ doses of BI-505 a sustained period of low-grade fever (19 days) and moderate, spontaneously resolving, C-reactive protein increase (15 days), without evidence of infection. CRP elevation was observed in 15% of patients (5/34) in the previous phase 1 study and might be a sign of secondary cytokine release [17]. Overall, BI-505 showed no clinically relevant efficacy on disease activity in the three evaluable patients with SMM, even if well tolerated.

The four patients in this study had a stable M component for over 2 years before enrollment (Table 1) and none of them have so far (November 2016; 6.4, 4.9, 5.0 resp. 10.4 years after diagnosis of SMM), progressed to symptomatic multiple myeloma. Several new prognostic variables are emerging making it possible to identify SMM patients at high risk for disease progression [39–41].

BI-505´s in vivo anti-myeloma activity was shown to be macrophage-dependent and was associated with significant recruitment of monocyte/macrophages to tumor lesions and myeloma-infiltrated bone marrow [16] (and unpublished data). BI-505´s anti-myeloma activity was found in early and disseminated experimental models of MM, or when combined with novel drugs e.g. the proteasome inhibitor bortezomib or the immune modulatory drug lenalidomide [16]. However, addition of lenalidomide or bortezomib to the treatment with BI-505 for patients with SMM, would imply a higher degree of expected toxicity, such as pancytopenia, susceptibility to infections and increased risk of secondary malignancies (lenalidomide) and peripheral neuropathy (bortezomib), counteracting the idea of a new treatment choice, without severe side effects for this patient category [41]. Recently published data on a clinical trial with carfilzomib/lenalidomide/dexamethasone to 12 patients with SMM reported that two patients developed secondary malignant neoplasms and one patient discontinued treatment due to a serious adverse event with grade 3 congestive heart failure [31].

Coming clinical trials will focus on exploring BI-505 activity in settings of lower tumor burden. A clinical phase II study exploring the addition of BI-505 to high dose melphalan and autologous stem cell transplantation has started recruiting patients (www.clinicaltrials.gov Identifier: NCT02756728).

## Supporting information

S1 Appendix Inclusion and exclusion criteria. (DOCX)

S2 Appendix TREND statement checklist. (PDF)

S3 Appendix Clinical study protocol. (PDF)
