# Supplementary material for: A single-arm, open-label, phase 2 clinical trial evaluating disease response following treatment with BI-505, a human anti-intercellular adhesion molecule-1 monoclonal antibody, in patients with smoldering multiple myeloma
Source: PLoS One. 2017 Feb 3;12(2):e0171205. doi: 10.1371/journal.pone.0171205 (PMC5291423; doi:10.1371/journal.pone.0171205)
Supplement: S1 Appendix — (DOCX) [file pone.0171205.s001.docx]

**S1 Appendix. Inclusion and Exclusion Criteria.**

**Inclusion Criteria**

- Diagnosis of smoldering multiple myeloma based on the IMWG criteria: Serum M protein level ≥3 g/100 ml and/or ≥10% bone marrow plasma cells AND absence of end-organ damage such as lytic bone lesions, anemia, hypercalcemia or renal failure that could be attributed to a plasma cell proliferative disorder
- Male or female, 18 years or older
- Ability to understand and willingness to sign an informed consent form
- Measurable disease, defined by a serum M protein of ≥1.0 g/100 ml
- Eastern Cooperative Oncology Group (ECOG) performance status 0 to 1
- Adequate hepatic function (aspartate transaminase [AST] and alanine transaminase [ALT] ≤2.5 times the upper limit of normal [ULN]; bilirubin ≤1.5 times the ULN)
- Adequate renal function (calculated serum creatinine clearance ≥ 50mL/min)
- Females of childbearing potential and males (and their partners): Agreement to use adequate contraception during the study and for at least 12 weeks after discontinuation. Adequate contraception: oral/systemic contraception, intrauterine device, last natural menstruation at least 24 months prior to baseline, surgical sterilization before baseline or hysterectomy prior to baseline
- No systemic corticosteroid use within 4 weeks prior to screening

**Exclusion Criteria**

- Diagnosis of symptomatic multiple myeloma or clinical suspicion of ongoing progression to symptomatic multiple myeloma
- Clinical findings indicating cardiac or renal amyloid light-chain (AL) amyloidosis
- Prior or current treatment with a proven or potential impact on myeloma cell proliferation or survival (including conventional chemotherapies, biological therapies, immunomodulatory drugs and proteasome inhibitors), as judged by the Investigator
- Use of any investigational agent within the last 3 months
- History of allogeneic stem cell or solid organ transplantation
- Prior malignancy in the last 2 years, with the exception of SMM, adequately treated basal cell or squamous cell carcinoma, cervical carcinoma in situ, prostate cancer Gleason < 6 and prostate-specific antigen (PSA) < 10 ng/mL, radically excised lobular or ductal carcinoma in situ (LCIS/DCIS) ≤ 15 mm breast cancer in women over 40 years old, or any malignancy for which the subject had undergone potentially curative therapy with no evidence of the disease for three years
- Evidence of significant active infection, requiring intravenous antibiotics, within 14 days before enrollment
- Current active infectious disease or positive serology for human immunodeficiency virus (HIV), hepatitis C virus (HCV) or hepatitis B surface antigen
- Patients with a history of cerebrovascular disease or atrial fibrillation were excluded (unless the event occurred more than two years previously and was adequately treated; and atrial fibrillation that was well controlled with medication)
- Patients with other severe conditions requiring treatment and close monitoring, e.g. cardiac failure of New York Heart Association (NYHA) grade > 3, unstable coronary disease or oxygen-dependent chronic obstructive pulmonary disease (CORP)
- Significant autoimmune disease requiring systemic treatment with corticosteroids or other immunosuppressive drugs during the previous 24 months (including rheumatoid arthritis, systemic lupus erythematosus, inflammatory bowel disease, psoriasis, multiple sclerosis, hemolytic anemia and glomerulonephritis, and other conditions that required such therapy (mild autoimmune phenomena and inactive disease were not exclusion criteria)
- Breast-feeding or positive pregnancy test
- Substance abuse or other concurrent medical conditions that could confound study interpretation or affect the patient´s ability to tolerate treatment or complete the study
