# Supplementary material for: A single-arm, open-label, phase 2 clinical trial evaluating disease response following treatment with BI-505, a human anti-intercellular adhesion molecule-1 monoclonal antibody, in patients with smoldering multiple myeloma
Source: PLoS One. 2017 Feb 3;12(2):e0171205. doi: 10.1371/journal.pone.0171205 (PMC5291423; doi:10.1371/journal.pone.0171205)
Supplement: S3 Appendix — (PDF) [file pone.0171205.s003.pdf]

Clinical Study Protocol

PRODUCT:
BI-505
BI PROTOCOL No:
12-BI-505-02
EudraCT No:
2012-004884-29
VERSION:
1.0
DATE:
5th December 2012

PROTOCOL TITLE:
A single-arm, Open-label, Phase 2 Clinical Trial Evaluating Disease Response Following Treatment With BI-505, a Human Anti–Intercellular Adhesion Molecule-1 Monoclonal Antibody, In Patients With Smoldering Multiple Myeloma

SPONSOR:
BioInvent International AB
Sölvegatan 41
S-223 70 Lund
Sweden

CRO:
TrialFormSupport
Box 165
SE-221 00 Lund
Sweden

SIGNATURE PAGE

COORDINATING INVESTIGATOR
Dr Markus Hansson, MD, PhD

5 Dec 2012

Signature Date

CLINICAL PROJECT MANAGER, BIOINVENT
Elisabeth Sonesson, PhD

5 Dec 2012

Signature Date

MEDICAL DIRECTOR, BIOINVENT
Magnus Korsgren, MD, PhD

5 Dec 2012

Signature Date

SENIOR BIOSTATISTICIAN
Anders Nordlund

Signature Date

SIGNATURE PAGE

COORDINATING INVESTIGATOR
Dr Markus Hansson, MD, PhD

Signature Date

CLINICAL PROJECT MANAGER, BIOINVENT
Elisabeth Sonesson, PhD

Signature Date

MEDICAL DIRECTOR, BIOINVENT
Magnus Korsgren, MD, PhD

Signature Date

SENIOR BIOSTATISTICIAN
Anders Nordlund

Anders Nordlund 05DEC 2012

Signature Date

Strictly confidential. This document contains information belonging to BioInvent International AB

STUDY CONTACTS

PRINCIPAL INVESTIGATORS

Markus Hansson, MD, PhD (COORDINATING INVESTIGATOR)
Department of Hematology
Skåne University Hospital, SE 221 85 Lund, Sweden
Tel: + 46 46 2220737 / +46 705 931110
E-mail: markus.hansson@med.lu.se

Morten Salomo, MD, PhD
Department of Hematology
Rigshospitalet, Blegdamsvej 3
DK-2100 København Ö, Denmark
Tel: +45 3545 19362
E-mail: morten.salomo@rh.regionh.dk

INVESTIGATIONAL SITES

1: Department of Hematology
Skåne University Hospital, SE 221 85 Lund, Sweden
2: Department of Hematology
Rigshospitalet, Blegdamsvej 3, DK-2100 København Ö, Denmark

MEDICAL CONTACT
Magnus Korsgren MD, PhD
Medical Director
BioInvent International AB
Sölvegatan 41, SE-223 70 Lund, Sweden
Tel: +46 46 286 2542 / +46 709 85 2534

SPONSOR REPRESENTATIVE:
Elisabeth Sonesson, PhD
Clinical Project Manager
BioInvent International AB
Sölvegatan 41, SE-223 70 Lund, Sweden
Tel: +46 46 286 8646 / +46 708 54 8646

BIOANALYTICAL ANALYSIS (serum BI-505, BI-505 immunogenicity)
Covance Clinical Pathology Services
Otley Road, Harrogate HD3 1PY, United Kingdom
Tel: +44 20 7910 7751
Fax: +44 20 7910 7804

BIOANALYTICAL ANALYSIS (PD)

BioInvent International AB
Sölvegatan 41, SE-223 70 Lund, Sweden
Tel: +46 46 286 8550
Fax: +46 46 211 0806

LABORATORY ANALYSIS

Klinisk Kemi, Labmedicin Skåne
Klinikgatan 19, SE-221 85 Lund, Sweden
Tel: +46 46 176030

Rigshospitalet, Klinisk Biokemisk Afdeling
Afsnit 3011, Blegdamsvej 9
2100 København Ø
Tel: +45 35453011

PHARMACOKINETIC ANALYSIS

BioInvent International AB
Sölvegatan 41, SE-223 70 Lund, Sweden
Tel: +46 46 286 8550
Fax: +46 46 211 0806

Strictly confidential. This document contains information belonging to BioInvent International AB

TABLE OF CONTENTS

1 Protocol Synopsis 8
2 Abbreviation and Definition of terms 11
3 Introduction 13
3.1 Multiple Myeloma 13
3.2 BI-505 13
3.3 Rationale for conducting this study 14
3.4 Benefit/Risk and Ethical assessment 15
4 Study Objectives 16
4.1 Primary Objectives 16
4.2 Secondary Objectives 16
4.3 Primary Endpoint 17
4.4 Secondary Endpoints 17
5 Study design 17
5.1 Overall study plan and procedures 17
5.2 Rationale for study design 21
6 Patient selection and withdrawal of patients 22
6.1 Screening 22
6.2 Inclusions criteria 22
6.3 Exclusion criteria 22
6.4 Withdrawal criteria 23
7 Study conduct 24
7.1 Restrictions during the study 24
7.2 Study treatment 24
7.2.1 BI-505 24
7.2.2 Storage and Handling 24
7.2.3 Drug Accountability and Compliance check 25
7.2.4 Administration of IMP 25
7.3 Concomitant therapy 25
8 Study assessments 26
8.1 Clinical Assessments 26
8.1.1 Demographics 26
8.1.2 Physical examination 26

8.1.3 Medical History ... 26
8.1.4 Vital Signs ... 26
8.1.5 Height and Body weight ... 27
8.1.6 ECG ... 27
8.2 Concomitant Medication and Therapy ... 27
8.3 Adverse Events ... 27
8.4 Laboratory Assessments ... 27
8.4.1 Clinical Laboratory Safety Tests ... 27
8.4.2 Pharmacokinetic Assessment ... 28
8.4.3 Antibodies against BI-505 ... 28
8.4.4 Serum M-protein and free light chains ... 28
8.4.5 Urinary M-protein ... 28
8.4.6 Bone marrow sampling ... 28
8.4.7 Blood PD Biomarkers ... 29
8.4.8 Blood DNA ... 29
9 Adverse events ... 29
9.1 Definitions ... 29
9.1.1 Study Period ... 29
9.1.2 Adverse Events ... 29
9.1.3 Serious Adverse Events ... 29
9.1.4 Adverse Drug Reaction ... 30
9.1.5 Unexpected Adverse Drug Reaction ... 30
9.1.6 Pregnancy ... 30
9.2 Documentation of adverse events ... 30
9.3 Classification of severity ... 30
9.4 Classification of relation to investigational medicinal product ... 30
9.5 Reporting of serious adverse events ... 31
9.6 Reporting of Suspected Unexpected Serious Adverse Reactions ... 31
9.7 Follow-up of adverse events ... 32
9.8 Abnormal Laboratory Values/Vital Signs ... 32
9.9 Emergency procedures ... 32
9.10 Indemnification ... 32
10 Statistical analysis ... 32
10.1 Sample size ... 33
10.2 Statistical analysis plan ... 33

Strictly confidential. This document contains information belonging to BioInvent International AB

10.3 Datasets 33
10.4 Patient disposition 33
10.5 Protocol deviations 33
10.6 Demographics and baseline characteristics 33
10.7 Treatment compliance and extent of exposure 33
10.8 Pharmacokinetic analysis 33
10.9 Efficacy analysis 34
10.10 Safety variables 34
10.10.1 Adverse Events 34
10.10.2 Laboratory Variables 35
10.10.3 Vital Signs 35
10.10.4 ECG Data 35
10.10.5 Physical Examination 35
11 Data handling 36
11.1 Electronic case report forms 36
11.2 Archiving of study documents on site 36
12 Monitoring and quality assurance (QA) 36
12.1 Compliance with good clinical practice 36
12.2 Access to source data and documentation 37
12.3 Monitoring 37
12.4 Source data verification 37
12.5 Audit and inspections 37
13 Stopping rules/discontinuation criteria 37
14 Ethics and regulatory review process 38
14.1 Ethical and regulatory conduct of the study 38
14.2 Financing 39
14.3 Protocol amendments 39
14.4 Patient information and consent 39
14.5 Confidentiality 39
14.6 Insurance 40
14.6.1 Letter of Indemnity 40
15 Publication 40
16 Appendix 41
17 References 41

1 PROTOCOL SYNOPSIS

**Study Title**	A single-arm, Open-label, Phase 2 Clinical Trial Evaluating Disease Response Following Treatment With BI-505, a Human Anti–Intercellular Adhesion Molecule-1 Monoclonal Antibody, In Patients With Smoldering Multiple Myeloma
**Sponsor**	BioInvent International AB<br>Sölvegatan 41<br>S-223 70 Lund, Sweden
**Coordinating Investigator**	Dr Markus Hansson, MD, PhD
**Investigational Sites**	Skåne University Hospital, Lund, Sweden<br>Copenhagen University Hospital, Copenhagen, Denmark
**Study Timelines**	January 2013 – March 2014
**Name of Product**	BI-505
**Objectives**	Primary<br>• To assess the tumor response rate (defined according to the IMWG uniform response criteria)<br>Secondary<br>• To further assess clinical safety of BI-505<br>• To further assess the pharmacokinetic profile of BI-505<br>• To further assess the pharmacodynamics of BI-505<br>• To further assess the immunogenicity profile of BI-505
**Study Design**	Phase II, multi-center, open-label, non-randomized, single-arm trial
**Duration**	50 days with a possibility of extended treatment up to 176 days in patients who respond to therapy
**Number of Subjects**	4 to 10 patients
**Methodology**	Following a screening period of up to 14 days eligible patients will receive five intravenous infusions of BI-505 over a seven weeks period (cycle 1): 3 mg/kg BW of BI-505 on treatment day 1, 10 mg/kg BW of BI-505 on treatment day 8, followed by three infusions with 10 mg/kg BW of BI-505 every second week. Patients are evaluated for disease activity based on data collected on treatment day 50. Patients with at least a minimal response will continue to the next dosing cycle (cycle 2) encompassing three bi-weekly intravenous doses of BI-505 (10 mg/kg BW). Patients who complete cycle 2 and have at least a partial response on treatment day 92 will continue to the next dosing cycle (cycle 3) including three additional bi-weekly intravenous doses of BI-505 (10 mg/kg BW). Disease activity, measured as M-protein levels in serum/urine, will be determined at regular intervals throughout the study. Patients with a complete response will receive three additional doses of BI-505 (10 mg/kg BW) before they end the study.<br>All patients will do an End of Study/Early Termination visit 4 weeks after the last dosing with BI-505. All patients will remain at the clinical unit for 10 hours after start of the first and second infusion, during which they are closely monitored for any untoward reactions. At subsequent infusions, patients will be observed for at least three hours after start of the infusions.
**Criteria for Evaluation**	Pharmacodynamics:<br>Blood/urine and bone marrow samples will be obtained at different time

	points throughout the course of the study for evaluation of disease activity.<br><br>Pharmacokinetics:<br>PK measurements will be performed before and after administration of the first dose of BI-505, and then at specific time points throughout the study.<br><br>Safety:<br>The safety evaluation will include blood pressure, pulse rate, body temperature, 12-lead electrocardiograms, clinical laboratory tests (hematology, serum biochemistry, and urinalysis), immunogenicity (anti-therapeutic antibodies), physical examination, and adverse events (according to CTCAE version 4.0) throughout the study.
**Inclusion Criteria**	To be considered eligible to participate in this study, a patient must meet the following criteria:<br>1. Diagnosis of Smoldering multiple myeloma based on the International Myeloma Working Group criteria:<br>a. Serum M-protein greater than or equal to 3 g/dl and/or bone marrow plasma cells greater than or equal to 10 percent.<br>b. Absence of end-organ damage such as lytic bone lesions, anemia, hypercalcemia or renal failure that can be attributed to a plasma cell proliferative disorder.<br>2. Male or female, 18 years or older.<br>3. Ability to understand and willingness to sign an informed consent form.<br>4. Measurable disease defined by serum M-protein ≥1.0 g/dL.<br>5. ECOG Performance status of 0-1.<br>6. Adequate hepatic function with aspartate transaminase and alanine transaminase ≤2.5 times the ULN; bilirubin ≤1.5 times the ULN.<br>7. Adequate renal function with calculated serum creatinine clearance ≥50mL/min.<br>8. Females of childbearing potential and males (and respective partners) must use adequate contraception during the study and at least for 12 weeks after discontinuation. Adequate contraception is defined as oral/systemic contraception, intrauterine device, or had her last natural menstruation at least 24 months prior to baseline, or has been surgically sterilized prior to baseline, or has had a hysterectomy prior to baseline.<br>9. No concurrent systemic corticosteroid use within four weeks prior to screening.
**Exclusion Criteria**	To be eligible to participate in this study, a patient must not meet any of the following criteria:<br>1. Patients with a diagnosis of symptomatic multiple myeloma or a clinical suspicion of an ongoing progression into symptomatic multiple myeloma.<br>2. Prior or current treatment having a proven or potential impact on myeloma cell proliferation or survival (including conventional chemotherapies, biological therapies, immunomodulatory drugs, or proteasome inhibitors), as judged by the Investigator.

	3. Use of any investigational agent within the last 3 months.<br>4. Current active infectious disease or positive serology for Human Immunodeficiency Virus (HIV), Hepatitis C Virus (HCV), or Hepatitis B Surface Antigen.<br>5. History of allograft or solid organ transplantation.<br>6. Prior malignancy within 2 years, excluding smoldering multiple myeloma, adequately treated basal cell or squamous cell skin cancer, cervical carcinoma in situ, prostate cancer Gleason ≤ 6 and PSA < 10 ng/mL, radically excised LCIS/DCIS ≤ 15 mm breast cancer in women > 40 yr, or any malignancy for which subject has undergone potentially curative therapy with no evidence of that disease for three years, or for which the treating physician deems the subject to be at low risk for recurrence.<br>7. A history of cerebrovascular disease or atrial fibrillation unless cerebrovascular disease ≥ 2 years ago and adequately treated with statins, antihypertensive and antithrombotic therapy; or atrial fibrillation, well controlled with medication.<br>8. Severe other conditions requiring treatment and close monitoring, e.g. cardiac failure ≥ NYHA (New York Heart Association) grade 3, unstable coronary disease or oxygen-dependent COPD.<br>9. Clinical findings indicating cardiac or renal AL amyloidosis<br>10. Evidence of significant active infection, requiring intravenous antibiotics, within 14 days before enrollment<br>11. Substance abuse or other concurrent medical conditions that, in the investigator's opinion, could confound study interpretation or affect the patient's ability to tolerate or complete the study<br>12. Significant autoimmune disease requiring systemic treatment with steroids or other immunosuppressive drugs during the last 24 months. This includes rheumatoid arthritis, systemic lupus erythematosis, inflammatory bowel disease, psoriasis, multiple sclerosis, hemolytic anemia and glomerulonephritis or other condition that has required such therapy. Mild autoimmune phenomena or inactive disease are not exclusion criteria<br>13. Breast feeding women or women with a positive pregnancy test.
**Statistical Analyses**	Data will be presented with summary statistics as mean, standard deviation, median, minimum and maximum for continuous data and frequency and percentage for categorical data. Patient data will be listed by patient. Statistical programming and analyses will be performed using the SAS® system.

2 ABBREVIATION AND DEFINITION OF TERMS

Abbreviation	Explanation
**ADR**	Adverse Drug Reaction
**AE**	Adverse Event
**AL amyloidosis**	Amyloid Light-chain amyloidosis
**ALAT / ALT**	Alanine Transaminase (SGPT)
**APTT**	Activated Partial Thromboplastin Time
**ASAT / AST**	Aspartate Transaminase (SGOT)
**ATA**	Anti-therapeutic antibody
**AUC**	Area under the concentration-time curve from time zero to infinity
**%AUC**	Percentage of AUC obtained by extrapolation
**BioInvent**	BioInvent International AB
**BI-505**	Monoclonal Antibody *vs* ICAM-1
**bpm**	Beats per minute
**BW**	Body Weight
**CA**	Competent Authorities
**CL**	Systemic Clearance
**C_{max}**	Maximum Plasma Concentration
**COPD**	Chronic Obstructive Pulmonary Disease
**CPMP**	Committee for Proprietary Medicinal Products
**CR**	Complete Response
**CRF**	Case Report Form
**CRO**	Contract Research Organization
**CTCAE**	Common Terminology Criteria for Adverse Events
**CV**	Coefficient of Variation
**DNA**	Deoxyribonucleic acid
**EC**	European Commission
**ECG**	ElectroCardioGram
**ECOG**	Eastern Cooperative Oncology Group
**eCRF**	Electronic Case Report form
**EU**	European Union
**GCP**	Good Clinical Practice
**GMP / cGMP**	Good Manufacturing Practice / currentGMP
**HCG**	Human Chorionic Gonadotropin
**HCV**	Hepatitis C Virus
**HIV**	Human Immunodeficiency Virus
**IB**	Investigator’s Brochure
**ICAM**	Intercellular Adhesion Molecule
**ICH**	International Conference on Harmonization
**ICSR**	Integrated Clinical Study Report
**ID**	Identification
**IEC**	Independent Ethics Committee
**IFN-γ**	Interferon-gamma
**Ig**	Immunoglobulin
**IgA**	Immunoglobulin A
**IgG1**	Immunoglobulin G, isotype 1
**IgM**	Immunoglobulin M
**IL-1β**	Interleukin-1 beta
**IL-6**	Interleukin-6

**IL-8**	Interleukin-8
**IMP**	Investigational Medicinal Product
**IMWG**	International Myeloma Working Group
**INR**	The International Normalized Ratio
**LCIS/DCIS**	Lobular carcinoma in situ/Ductal Carcinoma in Situ
**MedDRA**	Medical Dictionary for Regulatory Activities
**mg**	milligram
**mL**	milliliter
**MR**	Minimal Response
**mm**	millimeter
**N**	Numbers
**NYHA**	New York Heart Association
**PCR**	Polymerase Chain Reaction
**PD**	Pharmacodynamics
**PK**	Pharmacokinetics
**PR**	Partial Response
**PSA**	prostate-specific antigen
**QA**	Quality Assurance
**qs**	quantum sufficit
**RBC**	Red Blood Cell
**sec**	second
**SAE**	Serious Adverse Event
**SAP**	Statistical Analysis Plan
**SAS**	Statistical Analysis Software
**SD**	Standard Deviation
**SMM**	Smoldering (asymptomatic) Multiple Myeloma
**SUSAR**	Suspected Unexpected Serious Adverse Event
$t_{1/2}$	Elimination half-life
**TFS**	TrialFormSupport
**Tmax**	Time at maximum concentration
**TNF-α**	Tumor necrosis factor alpha
**ULN**	Upper Limit of Normal
**US**	United States
V_{ss}	Volume of distribution at steady state
**WBC**	White Blood Cell
**WMA**	World Medical Association

3 INTRODUCTION

3.1 MULTIPLE MYELOMA

Multiple myeloma is a hematologic malignancy characterized by the clonal proliferation of plasma cells in the bone marrow and, usually, the presence of a monoclonal immunoglobulin (Ig) in the blood and/or urine. It is the second most common hematological malignancy, with over 20 000 new cases diagnosed every year in the United States and a similar incidence in Europe. The incidence is higher with increasing age, and the median age at diagnosis is 67 years.

The clinical spectrum of multiple myeloma ranges from an asymptomatic, slowly proliferative disease, defined as smoldering myeloma, to full-blown, symptomatic multiple myeloma with end-organ damage and an aggressive clinical course.

Smoldering multiple myeloma was first described as a distinct clinical entity in 1980, and makes up 10-15 % of all myeloma diagnoses overall (Kyle 1980, Blade 2010). The International Myeloma Working Group (IMWG) has defined smoldering multiple myeloma as a disorder in which the patient has a serum monoclonal protein level of 3 g per deciliter or more, or a proportion of plasma cells in the bone marrow of 10 % or more, but no end-organ damage, such as lytic bone lesions, anemia, hypercalcemia or renal failure (IMWG 2003, Kyle 2009). The large majority of patients with smoldering multiple myeloma will ultimately develop symptomatic multiple myeloma. For example, a recent study demonstrated that 73 % of smoldering myeloma patients will go on to develop symptomatic myeloma within 15 years (Kyle 2007). In various studies, the median time to progression has ranged between 2 and 5 years. Due to this risk of developing symptomatic myeloma, these patients need to be monitored, and the IMWG has recently published guidelines around this (Kyle 2010).

Currently, standard of care dictates that smoldering myeloma patients should not receive therapy outside clinical trials (Korde 2011). A large number of studies have been conducted, or are ongoing, to assess whether various types of intervention may have a beneficial effect on the disease. Both drugs used for treatment of symptomatic myeloma, such as melphalan, prednisolone, bisphosphonates, thalidomide, and lenalidomide, as well as new drugs, such as various monoclonal antibodies (anti-KIR, anti-CS1, anti-IL-6, anti-DKK1), have been/are being tested for treatment of smoldering myeloma (Korde 2011). Yet, no drugs have been approved for the treatment of smoldering myeloma, but the hope is to develop a drug that may either control the malignant clone of plasma cells, or could be used with the goal of cure (Korde 2011).

Patients with symptomatic multiple myeloma exhibit symptoms or signs of end-organ damage. The most common end-organ damage consists of skeletal destruction with painful fractures, most often in central weight-bearing locations such as the vertebral column (Kyle & Rajkumar 2004). Other common features are renal failure, hypercalcemia, and bone marrow insuffiency with anemia and thrombocytopenia. Each of these myeloma manifestations, when present, severely impairs the quality of life and shortens the life-span of affected patients. The diagnosis of symptomatic myeloma carries a grave prognosis with a median survival of four to six years with currently available therapy.

3.2 BI-505

Several independent observations indicate that ICAM-1 is an attractive target for immunotherapy of multiple myeloma. This cell-surface receptor is highly expressed and

Strictly confidential. This document contains information belonging to BioInvent International AB

involved in the pathogenesis of many human malignancies, including myeloma (Huang 1995, Huang 1993, Smallshaw 2004, Wang 2006, Johnson 1988, Grothey 1998, Maruo 2002, Roche 2003, Rosette 2005, Aalinkeel 2004, Horst 1991). BI-505 is a fully human, high affinity IgG_1 monoclonal antibody directed against ICAM-1 that was generated from a proprietary n-CoDeR® human antibody fragment library. BI-505 has significant and broad antimyeloma activity *in vitro* and *in vivo*. The antimyeloma activity of BI-505 has been evaluated in several animal models representing early and advanced stages of multiple myeloma. Mode of action studies *in vitro* and *in vivo* demonstrate the significant ability of BI-505 to confer both FcγR-dependent and FcγR-independent antitumor activity.

A lack of BI-505 cross-reactivity with ICAM-1 of known and relevant toxicology species has precluded the assessment of BI-505 safety in conventional animal toxicological studies. As an alternative, the nonclinical safety assessment is relying on the assessment of cytotoxic or immunomodulatory effects using normal human ICAM-1 expressing cells.

The ongoing phase 1 clinical study is a multicenter, open-label, nonrandomized, repeat-dose, dose-escalation study of BI-505 in patients with relapsed/refractory multiple myeloma. The study is a first-in-human trial in which tolerability, safety, pharmacodynamics, and pharmacokinetics of BI-505 is evaluated following intravenous administration at doses from 0.0004 – 20.0 mg/kg once every second week. To date, more than 30 patients have been exposed to BI-505. The drug has been well tolerated and a review of the data from this study has not raised any safety concerns preventing administration of BI-505 to patients in subsequent clinical trials. Preliminary data reported and entered from the ongoing phase 1 study in patients through September 19, 2012, is detailed in the Investigator's Brochure (IB).

3.3 RATIONALE FOR CONDUCTING THIS STUDY

BI-505 is being developed as a treatment of multiple myeloma. In this phase II trial, effects of BI-505 in patients with smoldering multiple myeloma will be studied. The diagnosis of asymptomatic, smoldering myeloma makes up approximately 10-15 % of all patients with multiple myeloma.

Patients with smoldering myeloma are regarded as suitable for investigation of new and early intervention strategies for multiple myeloma (Korde 2011). This may be particularly relevant when studying therapeutic antibodies, since their effects are dependent on a functional immune system, including competent effector cells, such as natural killer cells and macrophages. Opposite to relapsed and/or refractory multiple myeloma patients who have been treated with chemotherapy and immune-modulatory drugs, the effector cell function in smoldering multiple myeloma patients is believed to be intact.

Monoclonal antibodies, such as BI-505, offer the possibility of targeted therapy to eliminate the tumor cells selectively with minimal side effects. Thus, this treatment modality is judged to be especially suitable for early treatment in patients, such as patients with smoldering myeloma, where severe side-effects are not acceptable from a risk-benefit perspective.

A number of monoclonal antibodies are currently in clinical trial for the treatment of myeloma. Most of the antibodies tested to date have not been efficacious in relapsed or refractory multiple myeloma patients with a suppressed immune system caused by the advanced disease and prior therapy with chemotherapy. Thus, our hypothesis is that monoclonal antibody therapy with BI-505 will be most effective when given as an early intervention in patients with a functional immune system.

Strictly confidential. This document contains information belonging to BioInvent International AB

3.4 BENEFIT/RISK AND ETHICAL ASSESSMENT

BI-505 is being developed for multiple myeloma, which at present is an incurable disease with high unmet medical need. ICAM-1 constitutes a new, interesting therapeutic target considering its broad expression in myeloma tissue and its involvement in myeloma pathophysiological mechanisms. BI-505 has a strong potential for therapeutic effect in myeloma based on the robust anti-tumor effects in animal models.

The primary objective of this study is to evaluate the disease response, based on M-protein levels, following treatment with BI-505 in patients with smoldering multiple myeloma. The patients to be recruited may have no or limited individual benefit of participation in the study, but the data will give scientifically valuable information and will inform the potential use of BI-505 to treat patients with smoldering multiple myeloma in subsequent clinical trials.

Relevant preclinical studies, e.g., pharmacology, toxicology, safety pharmacology and PK studies have been performed and are summarized in the Investigator´s Brochure (IB).

This is the second study initiated in humans with BI-505. In an ongoing phase 1 study in relapsed/refractory patients with multiple myeloma, BI-505 was administered intravenously at doses from 0.0004 – 20.0 mg/kg once every second week. To date, more than 30 patients have been exposed to BI-505. The drug has been well tolerated and a review of the data from this study has not raised any safety concerns preventing administration of BI-505 to patients in subsequent clinical trials. Preliminary data reported and entered from the ongoing phase 1 study in patients through September 19, 2012, is detailed in the IB. The Sponsor will immediately notify the investigators if any additional safety information becomes available during the study.

The development of any adverse event, abnormality in safety or laboratory variables will be thoroughly examined and evaluated on an ongoing basis during this clinical trial. In particular, based on preclinical and clinical data generated to date and generic risks associated with biologics, the following potential risks have been considered:

- Treatment with monoclonal antibodies is commonly associated with infusion-related reactions. Such reactions, if they are transient and mild or moderate, normally do not preclude further treatment with these pharmaceuticals, but may lead to modifications of doses, infusion rates and premedication with antihistamines, anti-pyretic drugs and steroids. Infusion-related reactions, such as chills, flushing, increased temperature, and headache have been observed following administration of BI-505 to patients in the ongoing phase I study. The infusion-related reactions have been limited to the first infusion of BI-505, have usually been of mild to moderate intensity, and have been completely reversible. Based on the experience from the phase I study, the current study is designed to reduce the risk of infusion-related reactions. Thus, since infusion-related reactions in the phase I study have been limited to the first dose, the initial dose is lower (3 mg/kg) than subsequent doses (10 mg/kg). Furthermore, since infusion-related reactions were observed in the 3 mg/kg-group of patients in the ongoing phase I study, the first dose (3 mg/kg) in the planned study will be administered over a prolonged time period (4 hours) compared to the infusion time (2 hours) used when this dose was given in the ongoing phase I study. As a precaution, also the second dose (10 mg/kg) will be given over a prolonged time period (4 hours) compared to subsequent doses (2 hours). Accordingly, all patients will remain at the clinical unit for 10 hours after start of the first and second infusion, during which they are closely monitored for any untoward reactions

(frequent measurements of vital signs and observation for relevant symptoms). At subsequent infusions, patients will be observed for at least 3 hours after start of the infusions. In addition, patients will be followed up for AEs by a telephone contact the day after the first and second study drug administration. As an additional precaution, all patients will receive premedication with an antihistamine and paracetamol prior to each infusion. Paracetamol will also be given at the end of the infusions of BI-505. Any infusion-related reactions should be treated per standard medical practice, based on the judgment of the Investigator.

- Based on the physiological role of ICAM-1 in leukocyte migration, and on findings from preclinical and clinical trials with the murine anti-ICAM-1 monoclonal antibody, Enlimomab, there may be an increased risk of infectious events when targeting ICAM-1. Especially the incidence of pneumonia seemed increased (by approximately 2:1) in the Enlimomab-treated patients in some of the randomized studies (Salmela 1999, Sherman 2001). No infections attributed to BI-505 have been observed to date in the ongoing phase I study. However, in view of this potential risk, the present study will not be open for patients with recent infection. In addition, participating patients will be closely monitored for infections in the current study, and patients who develop significant infections during the treatment period will have their treatment stopped until they have recovered from the infection.
- When given to patients with acute ischemic stroke (Sherman 2001), the murine anti-ICAM-1 monoclonal antibody Enlimomab resulted in an impaired functional recovery from neurological symptoms compared to placebo, and the negative effect was most evident among patients with atrial fibrillation. Immunogenicity of the murine antibody or neutrophil activation secondary to complement fixation has been suggested explanations, but with regard to the remaining uncertainty of the negative causative factors of Enlimomab in human stroke, this study is not open for patients with cerebrovascular disease less than 2 years ago or with uncontrolled atrial fibrillation.

The study design chosen for this study can be considered to minimize the safety risks, while optimising the possibilities to detect a clinically relevant effect of BI-505. The risk/benefit balance of using BI-505 in the present study in patients with smoldering multiple myeloma can therefore be considered acceptable.

4 STUDY OBJECTIVES

4.1 PRIMARY OBJECTIVES

- To assess the tumor response rate (defined according to the IMWG uniform response criteria)

4.2 SECONDARY OBJECTIVES

- To further assess clinical safety of BI-505
- To further assess the pharmacokinetic profile of BI-505
- To further assess the pharmacodynamics of BI-505
- To further assess the immunogenicity profile of BI-505

4.3 PRIMARY ENDPOINT

- Tumor response rate according to the IMWG criteria assessed by measurements of serum and urinary M-protein, and bone marrow plasma cells

4.4 SECONDARY ENDPOINTS

- Clinical safety parameters (Adverse events, clinical laboratory tests, ECG and vital signs)
- Pharmacokinetic parameters (AUC_t, $\%AUC_{ex}$, CL, C_{max}, t_{max}, $t_{½}$, V_{ss})
- Immunogenicity of BI-505 by measuring antibodies against BI-505
- Pharmacodynamic parameters (including ICAM-1 saturation on bone marrow plasma cells)

5 STUDY DESIGN

5.1 OVERALL STUDY PLAN AND PROCEDURES

This is a single-arm, open-label, phase II study, designed to assess the disease response following treatment with the monoclonal human antibody BI-505, when administered to patients with smoldering multiple myeloma.

Following a screening period of up to 14 days eligible patients will receive five intravenous infusions of BI-505 over a seven weeks period: 3 mg/kg BW of BI-505 on treatment day 1, 10 mg/kg BW of BI-505 on treatment day 8, followed by three infusions with 10 mg/kg BW of BI-505 every second week. This is considered one dosing cycle of 50 days (Fig. 1).

Patients are evaluated for disease activity based on data collected on treatment day 50. Patients with at least a minimal response based on M-protein levels will continue to the next dosing cycle (cycle 2) encompassing three bi-weekly intravenous doses of BI-505 (10 mg/kg BW). Patients who complete cycle 2 and have at least a partial response on treatment day 92 will continue to the next dosing cycle (cycle 3) including three additional bi-weekly intravenous doses of BI-505 (10 mg/kg BW). Disease activity, measured as M-protein levels in serum/urine, will be determined at regular intervals throughout the study according to the IMWG criteria (Durie 2006). A minimal response (MR) is defined as a ≥25 % but ≤49 % reduction of serum M-protein levels. Patients with a complete response will receive three additional doses of BI-505 (10 mg/kg BW) before they end the study. All patients will do an End of Study/Early Termination visit 28 days after last treatment, regardless of when treatment is stopped.

All patients will remain at the clinical unit for 10 hours after start of the first and second infusion, during which they are closely monitored for any untoward reactions. After subsequent infusions, patients will be observed for at least three hours after start of the infusions.

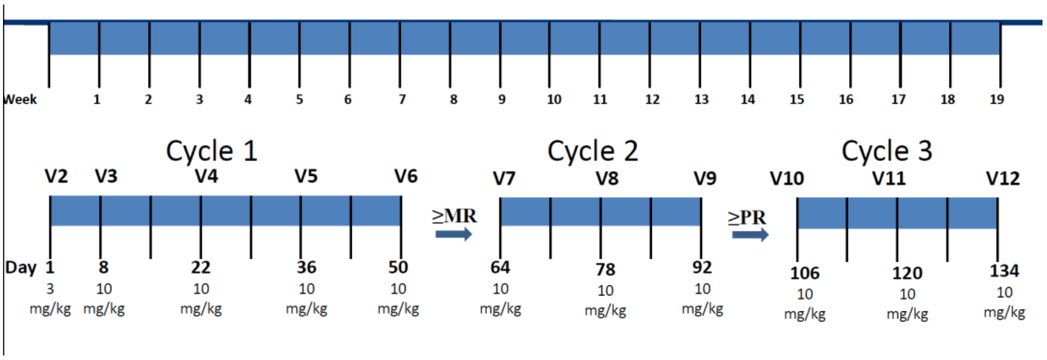

Figure 1. Overall Study Design. All patients will receive five intravenous infusions of BI-505 over a seven weeks period (cycle 1): 3 mg/kg BW of BI-505 on treatment day 1, 10 mg/kg BW of BI-505 on treatment day 8, followed by three infusions with 10 mg/kg BW of BI-505 every second week. Patients are evaluated for disease activity based on data collected on treatment day 50. Patients with at least a minimal response (MR) will continue to the next dosing cycle (cycle 2) encompassing three bi-weekly intravenous doses of BI-505 (10 mg/kg BW). Patients who complete cycle 2 and have at least a partial response (PR) on treatment day 92 will continue to the next dosing cycle (cycle 3) including three additional bi-weekly intravenous doses of BI-505 (10 mg/kg BW). Disease activity, measured as M-protein levels in serum/urine, will be determined at regular intervals throughout the study. Patients with a complete response (CR) will receive three additional doses of BI-505 (10 mg/kg BW) before they end the study. All patients will do an End of Study/Early Termination visit 4 weeks after the last dosing with BI-505.

Strictly confidential. This document contains information belonging to BioInvent International AB

STUDY FLOWCHART

	Screening	Treatment Period											End of study/Early Termination
		Cycle 1					Cycle 2 [a]			Cycle 3 [b]			
**Treatment Day**	**–14 to –2**	**1**	**8**	**22**	**36**	**50**	**64**	**78**	**92**	**106**	**120**	**134**	**28 days after last dose**
**Visit** [c]	**1**	**2**	**3**	**4**	**5**	**6**	**7**	**8**	**9**	**10**	**11**	**12**	
Informed consent	x												
Study drug administration [d, e]		x	x	x	x	x	x	x	x	x	x	x	
Eligibility	x	x											
Demographic information	x												
Medical history	x												
Complete physical examination	x												x
Limited physical examination [f]		x					x			x			
Body weight	x	x					x			x			x
Height	x												
Vital signs [g]	x	x	x	x	x	x	x	x	x	x	x	x	x
Electrocardiogram	x												x
Serum pregnancy test	x												
Urine pregnancy test [h]		x	x	x	x	x	x	x	x	x	x	x	x
Chemistry [h, i], Hematology [h, j]	x	x [k]	x	x		x	x		x	x		x	x
Urinalysis [h, l]	x	x [k]										x	x
Viral Serology	x												
Serum PK		x [m]	x [n]	x [m]	x [m]	x [m]	x [m]			x [m]		x [m]	x
Antibodies against BI-505		x [h]											x
Blood PD biomarkers [h]	x	x		x	x	x	x	x	x	x	x	x	x
Blood DNA	x												
Serum M-protein/free light chains [h]	x	x		x	x	x	x	x	x	x	x	x	x
Urinary M-protein	x				x [o]	x [o]	x [o]	x [o]	x [o]	x [o]	x [o]	x [o]	x [o]
Concomitant medications	x	x	x	x	x	x	x	x	x	x	x	x	x
Adverse events	x	x [p]	x [p]	x	x	x	x	x	x	x	x	x	x
Bone marrow sample [q]	x					x							

PD = pharmacodynamic; PK = pharmacokinetic

[a] Patients are evaluated for disease activity based on data collected on treatment day 50. Patients with at least a minimal response will continue to the next dosing cycle (cycle 2).

[b] Patients who complete cycle 2 and have at least a partial response on treatment day 92 will continue to the next dosing cycle (cycle 3).

[c] A ±3 days window is allowed for all visits except for visit 3 where the visit window is ±2 days.

[d] Patients will remain at the clinical unit for 10 hours after start of the first and second infusion, during which they are closely monitored for any untoward reactions. At subsequent infusions, patients will be observed for at least three hours after start of the infusion.

[e] Patients will receive premedication prior to each infusion. In addition, paracetamol will be administered at the end of each infusion. For details, see section 7.3.

[f] Includes evaluation of head, eye, ear, nose, and throat and cardiovascular, respiratory, and dermatological examinations.

[g] Includes systolic and diastolic blood pressure, pulse, and body temperature; to be obtained before, and at specific time points during and after each administration of study drug. Blood pressure determinations should be made with use of the same arm, after the patient has rested for 10 minutes. For details, see section 8.1.4.

[h] Samples will be collected 0–60 minutes prior to study drug administration.

[i] Includes sodium, potassium, glucose, creatinine, total calcium, total bilirubin, albumin, ALT, AST, alkaline phosphatase, C-reactive protein.

[j] Includes hemoglobin, hematocrit, platelet count, red blood cell (RBC) count, white blood cell (WBC) count, and absolute differential count (neutrophils, bands, eosinophils, lymphocytes, monocytes, basophils, and other cells), aPTT, INR.

[k] Results from Hematology, Chemistry, and Urinalysis at screening can be used as pre-dose data if sampled ≤ 3 days prior to first dosing.

[l] If positive dipstick, complete urinalysis with urine culture or/and microscopic examination,.

[m] Samples will be collected 0–60 minutes prior to and 30 minutes after the end of the infusion.

[n] Samples will be collected 0–60 minutes prior to and 30 minutes, 2 hours and 6 hours after the end of the infusion.

[o] 24 hour urine M-protein quantification, only for patients with a response (at least MR).

[p] Patients will be followed up for AEs by a telephone contact the day after the first and second study drug administration.

[q] In addition to the bone marrow sampling at screening and treatment day 50, a bone marrow sample should be collected from patients with at least a minimal response based on M-protein results, to confirm the response/verify ICAM-1 saturation.

5.2 RATIONALE FOR STUDY DESIGN

This is a single-arm, open-label phase II study designed to assess the disease response following treatment with the monoclonal human antibody BI-505, when administered to patients with smoldering multiple myeloma. The rationale for conducting this study in patients with smoldering myeloma is given in section 3.3.

The sample size has been estimated to minimize the number of patients exposed to BI-505 whilst obtaining sufficient information to assess effects on tumor burden.

The doses selected for this study are supported by safety, pharmacokinetic, and pharmacodynamic data from the ongoing phase 1 study in patients with relapsed/refractory multiple myeloma (for details, see IB, section 6). Notably, the doses in the planned study have been chosen to reduce the risk of infusion-related reactions. Thus, since infusion-related reactions in the phase I study have been limited to the first dose, the initial dose is lower (3 mg/kg) than subsequent doses (10 mg/kg). Furthermore, since infusion-related reactions were observed in the 3 mg/kg-group of patients in the ongoing phase I study, the first dose (3 mg/kg) in the planned study will be administered over a prolonged time period (4 hours) compared to the infusion time (2 hours) used when this dose was given in the ongoing phase I study. As a precaution, also the second dose (10 mg/kg) will be given over a prolonged time period (4 hours) compared to subsequent doses (2 hours). Accordingly, all patients will remain at the clinical unit for 10 hours after start of the first and second infusion, during which they are closely monitored for any untoward reactions (frequent measurements of vital signs and observation for relevant symptoms). At subsequent infusions, patients will be observed for at least 3 hours after the start of infusions. In addition, patients will be followed up for AEs by a telephone contact the day after the first and second study drug administration. As an additional precaution, all patients will receive premedication with an antihistamine and paracetamol prior to each infusion.

Based on the experience form the phase I study, dosing with 3 mg/kg BW of BI-505 will lead to complete saturation of all ICAM-1 epitopes on the patient´s bone marrow myeloma cells during the first dosing interval (one week). The drug concentration before next dose has been estimated to be around 1 µg/ml. Similarly, dosing with 10 mg/kg BW will lead to complete saturation during the subsequent, two week dosing intervals.

Patients are evaluated for disease activity based on data collected on treatment day 50. This treatment duration is considered sufficient to show an effect on the primary and secondary variables selected for this study.

However, patients with at least a minimal response will continue to the next dosing cycle (cycle 2) encompassing three bi-weekly intravenous doses of BI-505 (10 mg/kg BW). Patients who complete cycle 2 and have at least a partial response on treatment day 92 will continue to the next dosing cycle (cycle 3) including three additional bi-weekly intravenous doses of BI-505 (10 mg/kg BW). This design was chosen to optimize the chance to determine the maximum effect in each responding patients, whilst limiting the drug exposure in patients not responding to treatment. The decision to continue treatment must be made after careful evaluation of the risk/benefit ratio for each patient.

6 PATIENT SELECTION AND WITHDRAWAL OF PATIENTS

6.1 SCREENING

All patients considered for this study must give their written consent before any study-specific procedure is performed. Screening of a patient can take place up to 14 days before the first dosing on study day 1. To be eligible for the study, patients have to meet all inclusion criteria and must not meet any exclusion criteria.

6.2 INCLUSIONS CRITERIA

To be considered eligible to participate in this study, a patient must meet the following criteria:

1. Diagnosis of Smoldering multiple myeloma based on the International Myeloma Working Group criteria:
    a. Serum M-protein greater than or equal to 3 g/dL and/or bone marrow plasma cells greater than or equal to 10 percent.
    b. Absence of end-organ damage such as lytic bone lesions, anemia, hypercalcemia or renal failure that can be attributed to a plasma cell proliferative disorder.
2. Male or female, 18 years or older.
3. Ability to understand and willingness to sign an informed consent form.
4. Measurable disease defined by serum M-protein ≥ 1.0 g/dL.
5. ECOG Performance status of 0-1.
6. Adequate hepatic function with aspartate transaminase and alanine transaminase ≤ 2.5 times the ULN; bilirubin ≤ 1.5 times the ULN.
7. Adequate renal function with calculated serum creatinine clearance ≥ 50mL/min.
8. Females of childbearing potential and males (and respective partners) must use adequate contraception during the study and at least for 12 weeks after discontinuation. Adequate contraception is defined as oral/systemic contraception, intrauterine device, or had her last natural menstruation at least 24 months prior to baseline, or has been surgically sterilized prior to baseline, or has had a hysterectomy prior to baseline.
9. No concurrent systemic corticosteroid use within four weeks prior to screening.

6.3 EXCLUSION CRITERIA

To be eligible to participate in this study, a patient must not meet any of the following criteria:

1. Patients with a diagnosis of symptomatic multiple myeloma or a clinical suspicion of an ongoing progression into symptomatic multiple myeloma.
2. Prior or current treatment having a proven or potential impact on myeloma cell proliferation or survival (including conventional chemotherapies, biological therapies, immunomodulatory drugs, or proteasome inhibitors), as judged by the Investigator.
3. Use of any investigational agent within the last 3 months.
4. Current active infectious disease or positive serology for Human Immunodeficiency Virus (HIV), Hepatitis C Virus (HCV), or Hepatitis B Surface Antigen.
5. History of allograft or solid organ transplantation.

6. Prior malignancy within 2 years, excluding smoldering multiple myeloma, adequately treated basal cell or squamous cell skin cancer, cervical carcinoma in situ, prostate cancer Gleason ≤ 6 and PSA < 10 ng/mL, radically excised LCIS/DCIS ≤ 15 mm breast cancer in women > 40 yr, or any malignancy for which subject has undergone potentially curative therapy with no evidence of that disease for three years, or for which the treating physician deems the subject to be at low risk for recurrence.
7. A history of cerebrovascular disease or atrial fibrillation unless cerebrovascular disease ≥ 2 years ago and adequately treated with statins, antihypertensive and antithrombotic therapy; or atrial fibrillation, well controlled with medication.
8. Severe other conditions requiring treatment and close monitoring, e.g. cardiac failure ≥ NYHA (New York Heart Association) grade 3, unstable coronary disease or oxygen-dependent COPD.
9. Clinical findings indicating cardiac or renal AL amyloidosis.
10. Evidence of significant active infection, requiring intravenous antibiotics, within 14 days before enrollment.
11. Substance abuse or other concurrent medical conditions that, in the investigator's opinion, could confound study interpretation or affect the patient's ability to tolerate or complete the study.
12. Significant autoimmune disease requiring systemic treatment with steroids or other immunosuppressive drugs during the last 24 months. This includes rheumatoid arthritis, systemic lupus erythematosis, inflammatory bowel disease, psoriasis, multiple sclerosis, hemolytic anemia and glomerulonephritis or other condition that has required such therapy. Mild autoimmune phenomena or inactive disease are not exclusion criteria.
13. Breast feeding women or women with a positive pregnancy test.

6.4 WITHDRAWAL CRITERIA

Patients may be discontinued from study treatment and assessments at any time. Specific reasons for discontinuing a patient are:

- Voluntary discontinuation by the patient who is at any time free to discontinue his/her participation in the study, without prejudice to further treatment/care
- Safety reasons as judged by the investigator and/or the Sponsor
- Progression to symptomatic multiple myeloma or if there is a clinical suspicion of an ongoing progression into symptomatic multiple myeloma
- Severe non-compliance to protocol as judged by the investigator and/or the Sponsor
- Incorrect enrolment (i.e., the patient does not meet the required inclusion/exclusion criteria for the study)
- Patient lost to follow-up
- Pregnancy
- Dosing is delayed for more than 2 weeks, for any reason.

Strictly confidential. This document contains information belonging to BioInvent International AB

7 STUDY CONDUCT

7.1 RESTRICTIONS DURING THE STUDY

- Females of childbearing potential and males (and respective partners) must use adequate contraception during the study and at least for 12 weeks after discontinuation. Adequate contraception is defined as oral/systemic contraception, intrauterine device, or had her last natural menstruation at least 24 months prior to baseline, or has been surgically sterilized prior to baseline, or has had a hysterectomy prior to baseline.
- Patients should not donate blood or sperm at any time during the study or for 3 months following completion of the study.
- Patients should not take part in any other study whilst participating in this study.

7.2 STUDY TREATMENT

7.2.1 BI-505

BI-505 is a clear colorless, or slightly yellow liquid. It is intended for intravenous administration after dilution. BI-505 is formulated at 10 mg/mL, adjusted to pH 5.5 and supplied in 10 mL glass vials, containing 10 mL of BI-505 (10 mg/mL), i.e. a total of 100 mg BI-505.

Table 1: Investigational Medicinal Product

Ingredient	Quantity per mL	Function
BI-505 drug product	10 mg	Active ingredient
Sodium chloride	8.77 mg	Buffer
Sodiumacetate-3-hydrate	2.45 mg	Buffer
Acetic acid	0.12 µL	Buffer
Hydrochloric acid	qs ad pH 5.5	pH adjustment
Sodium hydroxide	qs ad pH 5.5	pH adjustment
Water for Injection	qs ad 1 mL	Diluent
Polysorbate 20	0.5 mg	Surfactant

BI-505 will be supplied to the site pharmacy in vials, as described above. Labeling will be carried out according to local requirements.

7.2.2 Storage and Handling

BI-505 vials should be kept dark at 2-8°C.

Detailed instructions for the preparation of BI-505, and for the labeling of infusion bags, will be provided in a Pharmacy manual. All handling and preparation of Investigational Medicinal Product (IMP) should take place at the pharmacy.

Strictly confidential. This document contains information belonging to BioInvent International AB

7.2.3 Drug Accountability and Compliance check

The investigator must ensure that a designated person receives study drug deliveries from Sponsor and that all such deliveries are:

- Recorded
- Handled and stored safely and properly
- Only dispensed to study patients according to the protocol
- Returned to BioInvent if unused or destroyed according to BioInvent's instructions

The investigator or designee must keep a drug inventory and accountability logs. The inventory will include details of the study drug received and dispensed to the patient, batch and ID numbers. Accounting must be made of any drugs deliberately or accidentally destroyed. Discrepancies between the amount of received and dispensed drug must be reconciled. Unused study drug must be kept and returned to Sponsor or destroyed according to the Sponsor's instructions after accountability by the study monitor. Disposal of hazardous material, e.g., syringes, needles, etc. must conform to applicable laws and regulations.

7.2.4 Administration of IMP

Patients will be given intravenous infusions of BI-505 in up to three cycles according to Fig 1.

On the first dosing occasion 3 mg/kg BW will be administered and at the following occasions 10 mg/kg BW will be administered.

BI-505 will be administered as an intravenous infusion (vein or central line) starting in the morning (preferably 08:00 – 11:00 am) by using an infusion pump. Each patient will be given the IMP diluted in saline during approximately 4 (dose 1 and 2) or 2 hours (subsequent doses). The infusion may be modified, e.g. paused and restarted, due to adverse reactions and the total infusion time will depend on any modifications during the infusion. The patient will be monitored during the infusion and body temperature, pulse rate and blood-pressure have to be recorded as indicated in Section 8.1.4. A nurse or other health-care professional must be in close vicinity during the infusion. The start and stop times and the total volume infused must be noted in the CRF to obtain a full dosing history. The patient is kept for observation for 10 hours after the start of the first and second infusions and for at least three hours after the start of all subsequent infusions. The patient must be monitored closely for any AEs during the infusion and during the observation time.

Patients who develop significant infections during the treatment period should have their treatment delayed until they have recovered from the infection. If dosing is delayed for more than 2 weeks, for any reason, the patient should be withdrawn from the study (see section 6.4).

7.3 CONCOMITANT THERAPY

No approved or investigational multiple myeloma treatments (including regular use of systemic steroids) can be administered during treatment with BI-505. No other investigational drug may be used concomitantly with the study drug and the patients are not allowed to participate concurrently in any other clinical study.

Any treatment having a proven or potential impact on myeloma cell proliferation or survival (including conventional chemotherapies, biological therapies, immunomodulatory drugs, or proteasome inhibitors) as judged by the investigator, is not allowed. Bisphosphonate treatment of osteoporosis must remain stable from 12 weeks prior to screening until the completion of the study.

All patients should receive premedication with paracetamol and an antihistamine prior to each dosing with BI-505. Paracetamol should also be given at the end of the infusions of BI-505. The dose and type of premedication may be adjusted on an individual basis, as judged by the investigator. The pre-medication should be entered as concomitant medication in the Case Report Form (CRF).

Supportive therapy considered necessary for the patient's welfare may be given at the discretion of the Investigator. This includes analgesics, medications for constipation or diarrhea, etc.

8 STUDY ASSESSMENTS

8.1 CLINICAL ASSESSMENTS

The following section describes the methods of assessments and list data to be recorded in the eCRF. A detailed Study Flowchart is presented in Section 5.

8.1.1 Demographics

At screening, date of birth, race and sex will be recorded in the eCRF.

8.1.2 Physical examination

A full physical examination will be performed at screening, and at the End of Study/Early termination visit. The examination will include the evaluation of head, eye, ear, nose, and throat, cardiovascular, dermatological, musculoskeletal, respiratory, gastrointestinal, and neurological systems. A limited physical examination will be performed at visits 2, 7 and 10 and will include evaluation of head, eye, ear, nose, and throat and cardiovascular, respiratory, and dermatological examinations. Findings at the physical examinations before first administration of IMP will be reported as medical history and findings post administration of IMP will be reported as AEs.

8.1.3 Medical History

At screening, any relevant past or current diseases will be recorded in the eCRF. Furthermore the date of SMM diagnosis, immunoglobulin type, and light chain type will be recorded.

8.1.4 Vital Signs

Vital signs (blood pressure, including position, pulse, and body temperature) will be measured at all visits. At visit 2 and 3, blood pressure and pulse should be measured prior, during (every 15 minutes), and after study drug administration, as well as one, three, and six hours after end of infusion. At visit 4-11, blood pressure and pulse should be measured prior, during (every 30 minutes), and after study drug administration, as well as one hour after end of infusion.

At visit 2 and 3, body temperature should be measured prior, and after study drug administration, as well as one, three, and six hours after end of infusion. At visit 4-11, body temperature should be measured prior, and after study drug administration, as well as one hour after end of infusion.

Strictly confidential. This document contains information belonging to BioInvent International AB

8.1.5 Height and Body weight

Body weight will be measured at screening (visit 1), pre-dose at visit 2, 7, and 10, and at the End of Study/Early termination visit. The patient's most recently measured body weight will be used to calculate the dose. The height will be measured at screening.

8.1.6 ECG

A 12-lead ECG will be recorded at screening and at the End of Study/Early termination visit, after 10-minute rest in a supine position, using standard procedures at the clinic. The ECG recordings will be obtained at recommended paper speed, 50 mm/sec. ECG print-outs (if not archive proof, the print-outs should be copied) will be stored in the patient's medical records. The investigator will make an overall evaluation of the ECG and the results will be recorded in the eCRF as normal/abnormal. Any abnormalities will be specified.

8.2 CONCOMITANT MEDICATION AND THERAPY

Any medication and/or treatment other than the study drug are considered concomitant medication and will be recorded in the eCRF.

8.3 ADVERSE EVENTS

Reporting of adverse events must be done at all visits and following the telephone contacts as described in Section 9.

8.4 LABORATORY ASSESSMENTS

The samples listed below will be taken according to the Study flowchart in Section 5.1. The total blood volume will not exceed the volume comprised by the consent.

A detailed description of the procedures for sampling, handling, storage and shipment of laboratory samples and all materials such as test tubes and labels will be given in the laboratory manual. Samples will be analyzed by laboratories as indicated below.

8.4.1 Clinical Laboratory Safety Tests

The samples will be analyzed by local laboratories at each site. Results from Hematology, Chemistry, and Urinalysis at screening can be used as pre-dose data if sampled ≤ 3 days prior to first dosing.

- **Hematology**: Hemoglobin, hematocrit, platelet count, red blood cell (RBC) count, white blood cell (WBC) count, and absolute differential count (neutrophils, bands, eosinophils, lymphocytes, monocytes, basophils, and other cells), aPTT, INR.
- **Clinical chemistry**: Sodium, potassium, glucose, creatinine, total calcium, total bilirubin, albumin, ALT, AST, alkaline phosphatase, C-reactive protein.
- **Urine**: Urine analysis will be assessed by dipstick for protein, glucose, and occult blood. In case of clinically significant abnormalities, a complete urinalysis with urine culture and/or microscopic examination will be performed.
- **Pregnancy test:** For women of childbearing potential, serum will be analyzed for Human Chorionic Gonadotropin (HCG) at screening. A urine pregnancy test will be performed before each drug administration and at the End of Study visit.
- **Viral serology:** Human Immunodeficiency Virus (HIV), Hepatitis C Virus (HCV), and Hepatitis B Surface Antigen.

Strictly confidential. This document contains information belonging to BioInvent International AB

8.4.2 Pharmacokinetic Assessment

The time points for PK samples is always given as "Time after previous infusion" i.e. when the total infusion volume has been administered. The samples will be analysed by Covance Laboratories Ltd.

PK sampling time from end of infusion:

Visit 2, 4, 5, 6, 7, 10, and 12: 0-60 min prior to, and 30 minutes after the end of the infusion.

Visit 3: 0-60 min prior to, and 30 minutes, 2 hours, and 6 hours after the end of the infusion.

A final PK sample will be taken at the End of Study/Early termination visit.

The exact date and time of each PK blood sampling must be entered into the eCRF.

8.4.3 Antibodies against BI-505

Blood samples for analysis of antibodies against BI-505 will be drawn 0-60 min prior to study drug administration on visit 2, and at the End of Study/Early termination visit. The samples will be analyzed by Covance Laboratories Ltd.

8.4.4 Serum M-protein and free light chains

The samples will be analyzed by local laboratories at each site. The following variables will be recorded in the eCRF:

- Serum M-protein
- Serum free light chains
- Kappa/lambda ratio
- IgG, IgA, IgM concentrations

8.4.5 Urinary M-protein

The samples will be analysed by local laboratories at each site and the results will be recorded in the eCRF.

8.4.6 Bone marrow sampling

The pharmacodynamic profile of BI-505 treatment will be measured by assessment of the saturation of ICAM-1 on plasma cells in bone marrow aspirates/biopsies. Samples will be taken at screening, and at visit 6. In addition, a bone marrow sample should be collected from patients with at least a minimal response based on M-protein results, to confirm the response/verify ICAM-1 saturation. The relative levels of BI-505 bound versus free ICAM-1 cell surface receptors will be assessed using labeled BI-505 (BI-505*) and Enlimomab (Enlimomab does not compete with BI-505 for binding to ICAM-1), respectively. In the aspirate/biopsy samples, the bone marrow cells will be labeled with BI-505* and/or Enlimomab and additionally CD38, CD138, CD19 monoclonal antibodies allowing for gated analysis of BI-505 bound versus total cell surface ICAM-1 molecules by flow cytometry/histology. Samples will be analyzed at BioInvent. Additional pharmacodynamic biomarkers, which may include analysis of proteins and metabolites and other biomarkers, may also be analysed and the samples will be stored for up to 5 years.

From each bone marrow sample, a smear will be obtained for assessment of the proportion of plasma cells in the bone marrow. The smears will be analyzed by local laboratories at each site.

8.4.7 Blood PD Biomarkers

Blood samples will be collected from patients for analysis of IL-1β, IFN-γ, TNF-α, IL-6 and IL-8 as detailed in the laboratory manual. Additional pharmacodynamic biomarkers, which include analysis of proteins and metabolites, may also be analysed and the samples will be stored for up to 5 years. These specimens will be used for research purposes to identify biomarkers that are predictive of response to BI-505 treatment. Samples will be drawn at all visits except from visit 3. Samples will be analysed by BioInvent or at not yet identified laboratories.

8.4.8 Blood DNA

A blood sample will be taken at screening for the pharmacogenetic assessment of FcγR allelic variants. From this sample, blood cells will be collected for genomic DNA isolation. The samples may be processed using techniques such as kinetic thermocycling (PCR) and/or DNA sequencing to study the genotype profile of primarily FcγR's but possibly also additional genes known to be involved in multiple myeloma, and any other genes relative to treatment response. The samples will be stored for a maximum period of 5 years. All genetic analysis described in this section will only be done on patients giving their consent on a separate informed consent form. Samples will be analysed by BioInvent.

The pharmacogenetic information gathered through the analysis of specimens should improve treatment outcome by predicting which patients are more likely to respond to BI-505, predicting which patients are susceptible to developing adverse side effects and/or predicting which patients are likely to progress to more severe disease states.

9 ADVERSE EVENTS

The Sponsor delegates to TFS to handle reporting of Serious Adverse Events, to EudraVigilance, EU Competent authorities and Ethics Committees as required by the relevant regulations. The Investigators should immediately report all SAE-s to CRO Safety Officer, as described in section 9.5. The CRO will keep the Sponsor informed. All safety related information will be collected and processed promptly, to comply with regulatory requirements.

9.1 DEFINITIONS

9.1.1 Study Period

For the purpose of safety reporting, the study period is defined as the interval between the time of screening until study termination.

9.1.2 Adverse Events

Adverse events (AE) are defined as any untoward medical occurrence in a patient that appears or worsens during the course of study, which does not necessarily have a causal relationship with the study treatment. An AE can therefore be any unfavorable sign, symptom or disease.

9.1.3 Serious Adverse Events

Serious adverse events (SAE) are defined as any untoward medical occurrence or effect which at any dose:

1. Results in death
2. Is life threatening

3. Requires hospitalization or prolongation of existing hospitalization
4. Results in persistent or significant disability or incapacity
5. Is a congenital anomaly/birth defect
6. Is an important medical event

9.1.4 Adverse Drug Reaction

All noxious and unintended responses to a medicinal product related to any dose should be considered an adverse drug reaction (ADR). The phrase "responses to a medicinal product" means that a causal relationship between a medicinal product and an AE is possible and that there is no other clear explanation.

9.1.5 Unexpected Adverse Drug Reaction

An ADR, the nature or severity of which is not consistent with the applicable product information is considered an unexpected ADR.

9.1.6 Pregnancy

A pregnancy, with no adverse events reported, in a patient in the study, should be recorded as a non-serious adverse event on the Adverse Event Form as well as on the Pregnancy Form, and reported to TFS using the same timelines as those for SAEs.

The investigator must follow up on the outcome of the pregnancy and report it to TFS. If the outcome of a pregnancy is abnormal, the infant should be followed for six months.

9.2 DOCUMENTATION OF ADVERSE EVENTS

If several signs, symptoms or diagnostic abnormalities are clearly related to a medically defined diagnosis or syndrome, the diagnosis should be reported on the AE pages in the eCRF. All clearly related signs, symptoms and abnormal diagnostic procedures should be grouped together as a single diagnosis. Grouping into a medical diagnose should only be done if every component sign and symptom is a medically and clearly acknowledged component of the diagnosis by standard textbook of medicine. If any of the signs or symptoms does not fit into a classic pattern of the diagnosis or syndrome, a separate AE should be reported for each sign or symptom.

SAEs must be reported on an SAE report form and on the AE pages in the eCRF.

All AEs during the study will be recorded in the eCRF. For each sign, symptom or diagnosis, the Investigator will provide the following information: type of event, date initiated/observed, severity, action taken, seriousness, date stopped, outcome and relation to investigational medicinal product. With respect to grading of any AE or SAE the maximum intensity will be recorded.

9.3 CLASSIFICATION OF SEVERITY

All adverse events must be classified using Common Terminology Criteria for Adverse Events v 4.0 (CTCAE).

9.4 CLASSIFICATION OF RELATION TO INVESTIGATIONAL MEDICINAL PRODUCT

The relationship to the IMP of all adverse events will be categorized according to the following table:

Strictly confidential. This document contains information belonging to BioInvent International AB

**UNRELATED**	Clinical event with an incompatible time relationship to IMP administration and that could be explained by underlying disease or other drugs or chemicals or is incontrovertibly not related to the IMP.
**UNLIKELY**	Clinical event whose time relationship to IMP administration makes a causal connection improbable, but that could plausibly be explained by underlying disease or other drugs or chemicals.
**POSSIBLE**	Clinical event with a reasonable time relationship to IMP administration, but that could also be explained by concurrent disease or other drugs or chemicals.
**PROBABLE/ CERTAIN**	Clinical event with a reasonable time relationship to IMP administration and is unlikely to be, or cannot be, attributed to concurrent disease or other drugs or chemicals.

9.5 REPORTING OF SERIOUS ADVERSE EVENTS

All AEs that meet the criteria for SAEs require a completion of a Serious Adverse Event Report Form. All SAEs must also be recorded on the AE pages in the eCRF. This applies to all SAEs that occur in the time from screening until the last follow-up visit.

The SAE must be reported immediately by the Investigator, within one calendar day (24h) from time of awareness by telephone or fax to TFS Drug Safety, who in turn will notify the Sponsor. Any follow-up data will be detailed in a subsequent SAE form, which also must be reported to TFS Drug Safety. In case of SUSARs, TFS will inform all participating sites within the same timelines as the regulatory reporting.

The Investigator should take all appropriate measures to ensure the safety of the patients. He or she should follow up the outcome of any serious adverse events (clinical signs, laboratory values or other, etc.) until the return to normal or until consolidation of the patient's condition.

In case of any serious adverse events, the patient must be followed up until clinical recovery is complete and laboratory results have returned to normal, or until progression has stabilized. This may imply that follow-up will continue after the patient has left the study and that additional investigations may be requested by the Sponsor.

In case of any serious adverse event brought to the attention of the Investigator at any time after cessation of treatment with investigational medicinal product and considered by him/her caused by the study treatment with a reasonable possibility, this should be reported to the Sponsor and to the authorities, if appropriate.

SAE Reporting contact details:

TFS, Drug Safety

E-mail: Safety.tfs@tfscro.com (24 hours)
Fax: +46 46 280 19 19 (24 hours)
Phone: +46 46 280 18 00 (switchboard)

9.6 REPORTING OF SUSPECTED UNEXPECTED SERIOUS ADVERSE REACTIONS

The Sponsor is responsible for informing the regulatory authorities, the European Medicines

Agency and the ethics committees of any individual case report of SAEs that are determined to be reportable by the Sponsor (i.e. a SUSAR). The Investigator will ensure all relevant information is provided to the Sponsor to allow the Sponsor to meet their obligations to report the SUSAR to the CA and IEC. For a SUSAR that is fatal or life-threatening, this should be reported as soon as possible and not later than 7 days after the Sponsor was first advised, for any other SUSAR this should be within 15 days. The Sponsor has delegated the reporting of SUSAR's to TFS.

9.7 FOLLOW-UP OF ADVERSE EVENTS

All adverse events must be followed until they are resolved or the patient's participation in the study ends. Instructions for reporting changes in an ongoing adverse event during a patient's participation in the study are provided in the instructions that accompany the Adverse Event Case Report Forms. In addition, any unresolved serious adverse events and non-serious events requiring follow up, as assessed by the Investigator, when the patient leaves the study should be followed until they resolve or until the Investigator assesses them as "chronic" or "stable". Resolution of such events must be documented in the patient eCRF.

9.8 ABNORMAL LABORATORY VALUES/VITAL SIGNS

An asymptomatic abnormal laboratory/vital sign finding should only be reported as an AE if it is clinically significant, if it fulfills the criteria for an SAE or if it causes the subject to discontinue the study.

If an abnormal laboratory/vital sign value is associated with clinical signs and symptoms, the sign/symptom should be reported as an AE and the associated laboratory/vital sign result should be considered additional information.

9.9 EMERGENCY PROCEDURES

It is the responsibility of the Investigator to ensure the availability of facilities sufficient to handle emergency situations during the study.

9.10 INDEMNIFICATION

The Sponsor will indemnify the Investigator (and the institution where the study is conducted) against any claim for damages brought by a patient who suffers a Research Related Injury and shall reimburse the Investigator for the actual cost of diagnostic procedures and medical treatment necessary to treat a Research Related Injury. For purposes of this Agreement, the term **"Research Related Injury"** means physical injury caused by the Product or procedures prescribed in the Protocol which are different from the medical management the patient would have received if he had not participated in the Study.

10 STATISTICAL ANALYSIS

The analyses described in this section concern the data collected until and including the End of Study/Early termination visit.

Data will be presented by summary statistics and in patient data listings. Summary statistics will be mean, standard deviation, median, minimum, maximum and number of patients for continuous data and frequency and percentage for categorical data. Summary statistics will be given in total. All data collected will be listed by patient. Statistical programming and analyses will be performed using the SAS® system.

Strictly confidential. This document contains information belonging to BioInvent International AB

A baseline measurement is considered as the last measurement before first administration of dose of study drug.

In general, missing values will not be replaced (exceptions are given below).

10.1 SAMPLE SIZE

The sample size is not based on any formal power calculation but with the aim to minimize the number of patients exposed to BI-505 whilst obtaining sufficient information to assess effects on tumor burden. A minimum of 4 patients and a maximum of 10 patients will be enrolled. This sample size is considered appropriate for a study of this type and an IMP such as BI-505.

10.2 STATISTICAL ANALYSIS PLAN

Data will be presented using summary statistics and in data listings and no statistical analyses are planned for this study. Therefore, no statistical analysis plan (SAP) will be prepared. Any deviations from the protocol will be described and justified in the Integrated Clinical Study Report (ICSR).

10.3 DATASETS

The following datasets will be defined:

Safety analysis set:	All subjects who received at least one dose of BI-505
PK analysis set:	Any subjects who received at least one dose of BI-505 and have evaluable PK data as determined by the pharmacokineticist

With the exception of PK data, all summaries will be based on the Safety set.

10.4 PATIENT DISPOSITION

The number of patients withdrawn from the study together with the primary reason for discontinuation will be listed.

10.5 PROTOCOL DEVIATIONS

Any recorded protocol deviations will be listed.

10.6 DEMOGRAPHICS AND BASELINE CHARACTERISTICS

Demographic variables (age, sex and race), immunoglobulin type, and light chain type will be presented using summary statistics.

Medical/surgical histories will be listed including comments and coded fields.

10.7 TREATMENT COMPLIANCE AND EXTENT OF EXPOSURE

Dosing details for each infusion will be listed along with any recorded deviations from the planned dosing regimen.

10.8 PHARMACOKINETIC ANALYSIS

Pharmacokinetic (PK) parameters (AUC, % AUC_{ex}, C_{max}, T_{max}, CL, V_{ss} and $t_{1/2}$) will be calculated using non-compartmental or compartmental analysis.

Summary statistics on concentration raw data will be reported using N (number of observations included), N_{obs} (number of observations), mean (arithmetic), SD, minimum, median, maximum, and % coefficient of variation (CV). Nominal time, relative to dosing will be used for presentation of both pre- and post-dose concentration data. If the actual time elapsed from dosing deviates more than 20% from the nominal time elapsed from dosing, the concentration at that time point will be excluded from the summary statistics of concentrations (except for pre-dose concentrations). Such exclusions will be described in a footnote of the table(s) concerned.

Concentration values deviating from the rest of the profile, *i.e.* concentration values that are biologically and pharmacokinetically implausible, may be excluded from the summary statistics on concentrations and the PK parameter estimation at the discretion of the responsible pharmacokineticist. Any such exclusion will be described in a footnote of the table(s) concerned.

Plots of concentration-actual time data will be presented for each subject on linear and semi-logarithmic scales. Geometric mean for all concentration-nominal time profiles will be presented for all patients in one graph on a linear and semi-logarithmic scale. Additional graphical presentations of PK data may be added at the discretion of the PK scientist.

Summary statistics on pharmacokinetic parameters will be presented using geometric mean, geometric coefficient of variation (CV%), harmonic mean (only for $t_{1/2}$), median, minimum value (min), maximum value (max) and number of observations (N). The geometric statistics will not be presented for T_{max}, and $\%AUC_{ex}$.

10.9 EFFICACY ANALYSIS

The primary efficacy endpoint, tumor response rate according to the IMWG response criteria, as assessed by measurements of serum M-protein, urinary M-protein and bone marrow plasma cells will be presented with summary statistics, including both absolute values and change from baseline.

Both absolute values and change from baseline will be presented with summary statistics for the following secondary efficacy parameters:

- Antibodies against BI-505
- PD biomarkers (e.g. IL-1β, IFN-γ, TNF-α, IL-6, IL-8 and ICAM-1 saturation on bone marrow plasma cells)

Additional PD biomarkers will be listed only.

10.10 SAFETY VARIABLES

10.10.1 Adverse Events

AEs will be reported using the MedDRA system organ class and preferred term. Any AEs for which severity changes will only be counted once (i.e., unique AE) at its worst severity and relationship to IMP, will be presented. If the severity or relationship to the IMP of an AE is missing, a worst-case scenario will be assumed (i.e. it will be set to severe or probable/certain relationship).

Only treatment-emergent AEs (i.e. commencing after dosing with the IMP) will be included in the summary tables. All AEs will be included in the data listings.

IMP-related AEs will be defined as events considered to have a possible or probable/certain relationship to the IMP. AEs leading to withdrawal will be defined as events where the IMP was discontinued as a result of the AE.

The denominator used for the calculation of percentages will be the number of patients in the safety dataset.

An overall summary of AEs will be given as outlined in Table 3. Unique AEs will also be summarized by severity. AEs will also be listed by preferred term. A separate listing with deaths, other serious and significant AEs will also be given.

Table 3. Summary of Adverse events.

	Patients
AEs	
Unique AEs	
AEs leading to withdrawal	
AEs leading to death	
Patients with at least one AE	
SAEs	
Unique SAEs	
Patients with at least one SAE	
Related AEs	
Unique related AEs	
Patients with at least one related AE	

10.10.2 Laboratory Variables

Laboratory data will be be presented graphically as mean values and for each patient individually. Clinical chemistry and hematology values falling outside reference ranges will be listed separately together with all clinical chemistry and hematology laboratory parameters at that time point, respectively.

10.10.3 Vital Signs

Vital signs data will be listed only. Changes from baseline of more than ±20 mmHG for blood pressure and more than ±15 beats per minute for heart rate will be listed separately.

10.10.4 ECG Data

For all patients a listing containing all assessments where result was not ‘Normal’ will be generated. Any abnormalities will be specified.

10.10.5 Physical Examination

For all patients a listing containing all assessments where result was not ‘Normal’ will be generated.

11 DATA HANDLING

11.1 ELECTRONIC CASE REPORT FORMS

Electronic Case Report Forms (eCRFs) will be used to record data in this study. Some predefined data can be directly entered into the eCRF. This will be specified in the Location of Source Data form, prior to the study start. All other data recorded during the study will be kept in the patient's medical records at the involved hospitals. Data that are not recorded directly in the eCRF will be transcribed from the patient's medical records. The eCRFs should be completed in a timely manner.

The Investigator will permit monitoring, audits, IEC review and regulatory inspections and will provide direct access to source data/documents. The data obtained will be protected according to relevant national laws in the respective countries.

Data entered in the eCRFs will be verified against source data. The source data verification will be described in the monitoring plan. All changes to data will be documented in the electronic audit trail.

According to a data management plan the entered data will be checked for consistency and plausibility. Errors, omissions or questions will be sent as electronic queries within the eCRF to the investigational site for resolution. The completed original CRFs are the sole property of BioInvent and should not without written permission from BioInvent be made available in any form to third parties, except for authorized audits performed for BioInvent and for representatives of appropriate Health/Regulatory Authorities. After study completion each site will receive CDs with all eCRF study data for the site patients and the Sponsor will receive CDs with eCRF data from all sites.

11.2 ARCHIVING OF STUDY DOCUMENTS ON SITE

The investigator at each investigational site must make arrangements to store the essential study documents (as defined in Essential Documents for the Conduct of a Clinical Trial (ICH E6, Guideline for Good Clinical Practice)) including the Investigator Site File, until BioInvent informs the investigator, in writing, that the documents are no longer to be retained. In addition the investigator is responsible for archiving all relevant source documents so that the trial data can be compared against source data after completion of the study (e.g. in case of inspection from authorities).

The investigator is required to ensure the continued storage of the documents, even if the investigator, for example leaves the clinic/practice or retires before the end of the required storage period.

12 MONITORING AND QUALITY ASSURANCE (QA)

12.1 COMPLIANCE WITH GOOD CLINICAL PRACTICE

This Study Protocol is designed to comply with the Guideline E6 produced by the International Conference on Harmonization (ICH) on the topic Good Clinical Practice (GCP) and published by the European Agency for the Evaluation of Medicinal Products (CPMP/ICH/135/95) and U.S. Department of Health and Human Services, Food and Drug Administration as well as other relevant guidelines issued by ICH, primarily the efficacy guidelines.

Strictly confidential. This document contains information belonging to BioInvent International AB

12.2 ACCESS TO SOURCE DATA AND DOCUMENTATION

The Investigator should guarantee access to source documents for the monitor and auditors as well as for inspection by appropriate regulatory agencies, and the IEC, if required.

12.3 MONITORING

The monitor will visit the study site on a regular basis to ensure that the study is conducted and documented in accordance with this protocol, ICH GCP guidelines, regulatory requirements and any study specific documents such as CRF completion guidelines.

Monitoring visits will be conducted to confirm that e.g.:

- The investigational team is adhering to the study protocol
- Informed consent has been obtained from all participants
- AEs and concomitant medication have been reported as required
- Data are being accurately recorded in the CRFs
- IMP is being stored correctly and drug accountability is being performed on an on-going basis
- Facilities are, and remain, acceptable throughout the study
- The Investigator and the site are receiving sufficient information and support throughout the study

Moreover, during monitoring visits the data recorded in the CRFs, source documents and other study-related records will be compared against each other in order to ensure accurate data that reflect the actual existence of the patient in the study i.e. source data verification.

12.4 SOURCE DATA VERIFICATION

Source Documents are original documents, data and records (e.g. hospital records, clinical and office charts, laboratory notes, memoranda, patient files and records kept at the pharmacy, recorded data from automated instruments etc.). Source Data is considered all information in original records and certified copies of clinical findings, observations, or other activities in the study. Source Data are contained in Source Documents (original records or certified copies). The location of source documents will be registered on a form specifying where source data can be located e.g. medical record, CRF, lab reports etc.

12.5 AUDIT AND INSPECTIONS

Audits or inspections, including source data verification, may be performed by representatives of TFS, the Sponsor, a CA and/or an IEC.

13 STOPPING RULES/DISCONTINUATION CRITERIA

The Investigator may discontinue the study for medical reasons, prior to inclusion of the intended number of patients. At the discretion of the Investigator in collaboration with the Sponsor, the study may be discontinued for other reasons, prior to inclusion of the intended number of patients.

A premature discontinuation of the study can be decided by the Sponsor in the following cases:

- The study is not conducted in accordance with the procedures defined in the approved protocol (i.e. low rate of recruiting, protocol deviations, failure to ensure the quality of the data collected)
- If new information on the Investigational Product which results in changes in the risk/benefit assessment becomes available
- Conditions that may warrant termination of the study include, but are not limited to the following:
  - The discovery of an unexpected and significant or unacceptable risk to the patients enrolled in the study. For example, if the accumulated rate of severe or atypical infections among all patients in the study would significantly deviate from the rate expected in similar patients who have not been treated with BI-505, all study medication must be discontinued and the study be put on hold awaiting further safety analysis.
  - Failure of the Investigators to enter patients at an acceptable rate in the study as a whole
  - A decision on the part of BioInvent to suspend or discontinue development of the drug

The Ethics Committee(s) and Regulatory Authorities in participating countries must be informed about a premature discontinuation of the study. Reporting must be done in accordance with the required timelines for each country.

14 ETHICS AND REGULATORY REVIEW PROCESS

14.1 ETHICAL AND REGULATORY CONDUCT OF THE STUDY

The principles laid down by the World Medical Association (WMA) Declaration of Helsinki, the ICH guidelines for Good Clinical Practice (GCP), as well as the demands of national drug and data protection laws and other applicable regulatory requirements, will be followed.

The Sponsor or designee will report promptly to a CA and an IEC any new information, which may indicate an adverse effect, on the safety of the patients or the conduct of the study. The last visit of the last patient, to the clinic, will be considered the end of the study. Within 90 days of the end of the study, or within 15 days if the study is stopped prematurely, the Sponsor or designee will provide the IEC and CA with a declaration of the end of the clinical study. In addition, a summary of the clinical study report, e.g. the synopsis, will be provided when available but no later than one year after study completion.

Sponsor responsibilities:

The study will be performed in accordance with the official version of the Declaration of Helsinki and in compliance with the ICH E6 guideline for GCP as well as with the respective laws and regulations in the participating countries.

Investigators Responsibilities:

Investigators responsibilities are set out in section 4 of the ICH E6 guideline for Good Clinical Practice.

14.2 FINANCING

This study has been initiated by BioInvent International AB ("the Sponsor"). The Sponsor finances all aspects of the study as described in this protocol. A legally binding agreement will be made between the Sponsor and the participating Hospitals to specify the terms of the co-operation before starting screening.

Patients will be reimbursed upon request to compensate for expenses in connection to the study.

Relevant financial information part of the agreement between Sponsor and hospitals will be submitted to the IEC as part of the application. This includes the sum by which the hospitals are compensated by Sponsor for clinical activities performed as part of the conduct of this study, and any other important financial information.

14.3 PROTOCOL AMENDMENTS

Deviations from the protocol will not be permitted without the prior approval from the Sponsor, except in medical emergency situations. Any amendments to the protocol must be discussed and agreed with the Sponsor. Substantial amendments will be submitted to the relevant CA and IEC for review and approval, as specified in the EU regulations. Non-substantial amendments will be sent to the relevant EU competent authorities for notification, where required.

14.4 PATIENT INFORMATION AND CONSENT

The Principal Investigator will be responsible for ensuring that no patient undergoes any study-related examination or activity before that patient has given written informed consent to participate in the study. The written consent must be given by the patient, after he or she has been given detailed information about the study. The verbal explanation must cover all the elements specified in the written information provided to the patient.

The patient must be given every opportunity to clarify any points he does not understand and, if necessary, ask for more information. At the end of the interview, the patient will be given time to consider the study information. After the informed consent document has been signed, a copy will be given to the patient and the original will be archived in the Investigator site file.

It should be emphasized to the patient that he/she is free to withdraw from the study at any time. Patients who refuse to return or withdraw the written informed consent should not be included or continue in the study.

To ensure medical confidentiality and data protection, the signed informed consent forms must remain with the Investigator for at least 15 years after completion of the study. The Investigator will allow these to be inspected by authorized persons on request, and the Investigator or his assistant will confirm, by signing and dating, that informed consent is obtained.

14.5 CONFIDENTIALITY

Patients will be informed that all study findings and results will be electronically stored and handled with strict confidentiality only by authorized persons. During the study, patients will be identified by initials, and, after inclusion, patient number. In the study database patients will only be identified by initials, date of birth, and patient number.

All materials, information (oral or written) and unpublished documentation provided to the Investigators (or any company/institution acting on their behalf), inclusive of this protocol, the patient Case Report Forms and the Investigator's Brochure, are the exclusive property of the Sponsor and may not be given or disclosed, either in part or in whole, by the Investigator or by any person under his/her authority to any third party without the prior express consent of the Sponsor.

However, the submission of this protocol and other necessary documentation to the IEC is expressly permitted, the IEC members having the same obligation of confidentiality.

The Investigator shall consider all information, results, discoveries, records accumulated, acquired, or deduced in the course of the study, other than that information to be disclosed by laws, as confidential and shall not disclose any such results, discoveries, records to any third party without the Sponsor's prior written consent.

14.6 INSURANCE

Appropriate insurance cover has been taken out in favor of patients participating in clinical studies. The coverage is provided patient to the terms and conditions of the clinical study insurance. Insurance cover exists for health damages as a result of measures, which are carried out in connection with the clinical study on the active treatment. By the terms of insurance the Investigator as well as the Sponsor must adhere strictly to the study protocol and comply with the respective prevailing level of scientific state of the art. Patients must strictly adhere to the instructions provided by the Investigator or his assistant, and may only undergo other medical treatments after approval from the Investigator, except in cases of emergencies.

14.6.1 Letter of Indemnity

The Sponsor certifies that he has taken out a liability insurance policy which covers the liability of the Investigator and his/her co-workers and which is in accordance with local laws and requirements. Specific statements will be contained in an appendix where needed.

15 PUBLICATION

The Sponsor retains exclusive ownership of all data, results, reports, findings, discoveries and any other information collected during this study. Therefore, the Sponsor reserves the right to use the data from the present study either in the form of CRF (or copies of these), or in the form of a report, with or without comments and with or without analysis, in order to submit them to the health authorities of any country.

By signing this protocol, the Investigator agrees that the results of this study may be used for submission to national and/or international registration and supervising authorities. The authorities will be notified of the Investigator's name, address, qualifications and extent of involvement.

The Sponsor is committed to presenting or publishing the results of this study, including sub studies, both if the results are positive and negative. The presented or published data should be done using clean, checked and validated data only, in order to ensure the accuracy of the results. It is agreed that, before publication, BioInvent will be given the opportunity to review and comment upon the manuscript. The time for review should not exceed 60 days after receipt of the manuscript. If the principal investigator/investigators have not submitted the results for publication within 6 months after completion of the final ICSR, BioInvent will have the right to publish. In this case the principal investigator/investigators will be given 60 days to review and comment on the manuscript prior to submission to the publisher. It is agreed between the Principal Investigator/Co-Investigators and BioInvent that data from the study will be used by BioInvent in connection with the development of the study product. Information may therefore be disclosed as required to other Investigators, future partners to BioInvent and to Competent Authorities.

Sponsor may require any proposed publication or presentation to be delayed for up to three months to enable a patent application to be prepared and filed. The three month period shall commence on the date of receipt of the proposed publication or presentation, or from the date when all relevant data from the study are made available to Sponsor, whichever is later.

16 APPENDIX

- Common Terminology Criteria for Adverse Events v 4.0 (CTCAE)

17 REFERENCES

Aalinkeel R, Nair MP, Sufrin G, Mahajan SD, Chadha KC, Chawda RP, Schwartz SA. Gene expression of angiogenic factors correlates with metastatic potential of prostate cancer cells. Cancer Res 2004; 64: 5311-21.

Blade J, Dimopoulos M, Rosinol L, Rajkumar SV, Kyle RA. Smoldering (asymptomatic) multiple myeloma: Current diagnostic criteria, new predictors of outcome, and follow-up recommendations. J Clin Oncol 2010; 28: 690-697.

Durie BGM, Harousseau J-L, Miguel JS, Bladé J, Barlogie B, Anderson K, Gertz M, Dimopoulus M, Westin J, Sonneveld P, Ludwig H, Gahrton G, Beksac M, Crowley J, Belch A, Boccadoro M, Turesson I, Joshua D, Vesole D, Kyle R, Alexanian R, Tricot G, Attal M, Merlini G, Powles R, Richardson P, Shimizu K, Tosi P, Morgan G, and Rajkumar SV. International uniform response criteria for multiple myeloma. Leukemia. 2006; 20: 1467-1473.

Grothey A, Heistermann P, Philippou S, Voigtmann R. Serum levels of soluble intercellular adhesion molecule-1 (ICAM-1, CD54) in patients with non-small-cell lung cancer: correlation with histological expression of ICAM-1 and tumour stage. Br J Cancer 1998; 77: 801-807.

Horst E, Radaszkiewicz T, Hooftman-den Otter A, Pieters R, van Dongen JJ, Meijer CJ, Pals ST. Expression of the leucocyte integrin LFA-1 (CD11a/CD18) and its ligand ICAM-1 (CD54) in lymphoid malignancies is related to lineage derivation and stage of differentiation but not to tumor grade. Leukemia. 1991; 5: 848-853.

Huang YW, Burrows FJ, Vitetta ES. Cytotoxicity of a novel anti-ICAM-1 immunotoxin on human myeloma cell lines. Hybridoma 1993; 12: 661-675.

Strictly confidential. This document contains information belonging to BioInvent International AB

Huang YW, Richardson JA, Vitetta ES. Anti-CD54 (ICAM-1) has antitumor activity in SCID mice with human myeloma cells. Cancer Res 1995; 55: 610-616.

The International Myeloma Working Group. Criteria for the classification of monoclonal gammopathies, multiple myeloma and related disorders: a report of the International Myeloma Working Group. British Journal of Haematology 2003; 121: 749–757.

Johnson JP, Stade BG, Hupke U, Holzmann B, Riethmuller G. The melanoma progression-associated antigen P3.58 is identical to the intercellular adhesion molecule, ICAM-1. Immunobiology 1988; 178: 275-284.

Korde N, Kristinsson SU and Landgren O. Monoclonal gammopathy of undetermined significance (MGUS) and smoldering multiple myeloma (SMM): novel biological insights and development of early treatment strategies. Blood 2011; 117:5573-5581.

Kyle RA, and Greipp PR. Smoldering multiple myeloma. N Engl J Med 1980; 302: 1347-1349.

Kyle RA, and Rajkumar SV. Multiple myeloma. N Engl J Med 2004: 351:1860-1873.

Kyle RA, Remstein ED, Therneau TM, *et.al*. Clinical Course and Prognosis of Smoldering (Asymptomatic) Multiple Myeloma. N Engl J Med 2007; 356: 2582-90.

Kyle RA, Rajkumar SV. Criteria for diagnosis, staging, risk stratification and response assessment of multiple myeloma. Leukemia 2009; 23: 3-9.

Kyle RA, Durie BGM, Rajkumar SV *et.al*. Monoclonal gammopathy of undetermined significance (MGUS) and smoldering (asymptomatic) multiple myeloma: IMWG consensus perspectives risk factors for progression and guidelines for monitoring and management. Leukemia 2010; 24: 1121-1127.

Maruo Y, Gochi A, Kaihara A, Shimamura H, Yamada T, Tanaka N, Orita K. ICAM-1 expression and the soluble ICAM-1 level for evaluating the metastatic potential of gastric cancer. Int J Cancer 2002; 100: 486-490.

Roche Y, Pasquier D, Rambeaud JJ, Seigneurin D, Duperray A. Fibrinogen mediates bladder cancer cell migration in an ICAM-1-dependent pathway. Thromb Haemost 2003; 89: 1089-1097.

Rosette C, Roth RB, Oeth P, Braun A, Kammerer S, Ekblom J, Denissenko MF. Role of ICAM1 in invasion of human breast cancer cells. Carcinogenesis 2005; 26: 943-950.

Salmela K, Wramner L, Ekberg H, Hauser I, Bentdal Ø, Lins LE, Isoniemi H, Bäckman L, Persson N, Neumayer HH, Jørgensen PF, Spieker C, Hendry B, Nicholls A, Kirste G, Hasche G. A randomized multicenter trial of the anti-ICAM-1 monoclonal antibody (enlimomab) for the prevention of acute rejection and delayed onset of graft function in cadaveric renal transplantation: a report of the European anti-ICAM-1 renal transplantation study group. Transplantation 1999; 67: 729-736.

Sherman D, Clark W, Schneider D, Bes A. Enlimomab acute stroke trial investigators. Use of Anti-ICAM-1 therapy in ischemic stroke. Results of the Enlimomab acute stroke trial. Neurol 2001; 57: 1428-1434.

Smallshaw JE, Coleman E, Spiridon C, Vitetta ES. The generation and anti-myeloma activity of a chimeric anti-CD54 antibody, cUV3. J Immunother 2004; 27: 419-424.

Strictly confidential. This document contains information belonging to BioInvent International AB

U.S. Department of Health and Human Services, Food and Drug Administration, Center for Drug Evaluation and Research (CDER), Center for Biologics Evaluation and Research (CBER). Guidance for Industry – E6 Good Clinical Practice: Consolodated Guidance, April 1996 ICH/ European Agency for the Evaluation of Medicinal Products. Note for Guidance on Good Clinical Practice (CPMP/ICH/135/95), Approval 17 July 1996.

Wang S, Coleman EJ, Pop LM, Brooks KJ, Vitetta ES, Niederkorn JY. Effect of an anti-CD54 (ICAM-1) monoclonal antibody (UV3) on the growth of human uveal melanoma cells transplanted heterotopically and orthotopically in SCID mice. Int J Cancer 2006; 118: 932-941.
